# The Effectiveness of eHealth Interventions for Weight Loss and Weight Loss Maintenance in Adults with Overweight or Obesity: A Systematic Review of Systematic Reviews

**DOI:** 10.1007/s13679-023-00515-2

**Published:** 2023-06-24

**Authors:** Sakris K. E. Kupila, Anu Joki, Laura-U. Suojanen, Kirsi H. Pietiläinen

**Affiliations:** 1 Obesity Research Unit, Research Program for Clinical and Molecular Metabolism, Faculty of Medicine, University of Helsinki, Helsinki, Finland; 2 HealthyWeightHub, Endocrinology, Abdominal Center, Helsinki University Hospital and University of Helsinki, Helsinki, Finland

**Keywords:** eHealth, Digital health, Telemedicine, Obesity, Weight loss, Weight loss maintenance

## Abstract

**Purpose of Review:**

The purpose of this study is to evaluate the effectiveness of eHealth interventions for weight loss and weight loss maintenance among adults with overweight or obesity through a systematic review of systematic reviews.

**Recent Findings:**

This study included 26 systematic reviews, covering a total of 338 original studies, published between 2018 and 2023. The review indicates that eHealth interventions are more effective than control interventions or no care and comparable to face-to-face interventions. The effect sizes remain relatively small when comparing eHealth interventions to any control conditions, with mean differences of weight loss results from − 0.12 kg (95% CI − 0.64 to 0.41 kg) in a review comparing eHealth interventions to face-to-face care to − 4.32 kg (− 5.08 kg to − 3.57 kg) in a review comparing eHealth interventions to no care. The methodological quality of the included studies varies considerably. However, it can be concluded that interventions with human contact work better than those that are fully automated.

**Summary:**

In conclusion, this systematic review of systematic reviews provides an updated understanding of the development of digital interventions in recent years and their effectiveness for weight loss and weight loss maintenance among adults with overweight or obesity. The findings suggest that eHealth interventions can be a valuable tool for delivering obesity care to more patients economically. Further research is needed to determine which specific types of eHealth interventions are most effective and how to best integrate them into clinical practice.

**Supplementary Information:**

The online version contains supplementary material available at 10.1007/s13679-023-00515-2.

## Introduction

A range of health complications, including type 2 diabetes, cardiovascular disease, hypertension, sleep apnea, certain forms of cancer, and increased mortality, are associated with obesity [1]. According to the World Health Organization, more than one billion people worldwide are living with obesity, and the number is still increasing [2]. As such, reducing overweight and obesity is the key to public health.

Lifestyle changes, such as adopting a healthy diet and increasing physical activity, are known to be challenging but feasible for achieving long-term weight management or weight loss [3, 4]. Behavioral weight management interventions have been found to result in approximately 2–2.5 kg more weight loss than control conditions at the 12–18 month mark [5, 6]. However, the long-term success in maintaining weight loss is limited, with only about 20% of individuals estimated to keep the weight off for a year or more [7, 8]. Despite this, these interventions are crucial due to their additional advantages, such as preventing diabetes and premature mortality [9].

The multifactorial causes of obesity necessitate targeting different levels of contributing factors (e.g., social support, favorable environment, individual factors) in obesity treatment. However, there are several challenges in delivering these services in primary care. Weight counseling is perceived as laborious by health professionals, and there is a limited understanding of obesity care and uncertainty about how to initiate a discussion on weight and what is the appropriate terminology and language to use [10, 11]. Effective weight loss requires time, as frequent contact between healthcare professionals and patients is associated with greater weight loss outcomes [5]. Limited time and resources, including the organization of weight management groups or provision of constructive support, are frequently cited as obstacles to individual-level obesity treatment [10, 11].

Digital health, defined by the World Health Organization as the use of information and communication technologies for improving health, has been proposed as a solution to promote healthy lives and well-being for people of all ages [12]. This novel approach is also suitable for reducing obesity as it can recognize the complexity of obesity and provide patient-centered, multidisciplinary care that considers the personalized needs of individuals living with obesity. Digital health technologies also have the potential to reach a considerable number of people and improve access to obesity care as well as reduce the costs of healthcare systems as they are cost-effective [13, 14]. Furthermore, digital health tools are accessible to a wide range of people regardless of their location or physical abilities and are flexibly available in terms of time.

The COVID-19 pandemic expedited the need and development of healthcare provided in ways other than in person. The interest in eHealth grew, and new innovations emerged. This time also prompted a welcomed uprising in eHealth research. Obesity was early on recognized as a risk factor for COVID-19 complications [15]. Thus, digital weight loss interventions were of more interest than ever before.

Even prior to the pandemic, digital weight loss and weight maintenance programs were already gaining popularity. As more trials were conducted, numerous systematic reviews were published to conclude their effectiveness and components. This article aims to review the past five years of systematic reviews on eHealth weight loss and weight maintenance interventions to provide an updated understanding of the development of digital interventions in recent years. Our primary focus is to offer a comprehensive overview of the effectiveness of eHealth interventions for weight loss and weight loss maintenance among adults with overweight or obesity.

## Methods

We followed guidelines for conducting systematic reviews of systematic reviews suggested by Smith et al. [16] as well as the Preferred Reporting Items for Systematic reviews and Meta-Analyses (PRISMA) [17]. We followed a pre-defined, albeit not prospectively registered, protocol.

### Inclusion and Exclusion Criteria

We followed the PICOS (population, intervention, control, outcome, study design) framework when formulating our inclusion and exclusion criteria (Table 1). We defined eHealth interventions as interventions delivered via websites, mobile phone applications, or other kinds of online programs with electronic components. This excluded interventions delivered solely through, e.g., phone or video calls, short messaging services (SMS), electronic chats, social media groups, or email, or the use of wearable devices without an accompanying eHealth intervention. We also excluded digital tools used outside an eHealth intervention design (e.g., meal logging applications combined with standard care) and interventions without interactivity (e.g., information-only website). We included only interventions for weight loss or weight loss maintenance rather than interventions for treating any specific underlying disease (e.g., type 2 diabetes, metabolic syndrome, hypertension, cardiovascular risk factors, or cancer). We also excluded studies focusing on lifestyle change, rather than weight loss or weight loss maintenance (such as interventions focusing solely on increasing vegetable and fruit intake, reducing sedentary behavior, or increasing physical activity).

**Table 1:** Inclusion and exclusion criteria of the included reviews

	**Inclusion criteria**	**Exclusion criteria**
**Population**	Adults aged 18 or older with overweight or obesity (BMI ≥ 25 kg/m^2^, or BMI ≥ 23 kg/m^2^ in people of Asian ethnicity)	Pregnant people or those in the postpartum period; people who have had or are waiting for bariatric surgery; people waiting for other types of surgery
**Intervention**	eHealth weight loss or weight loss maintenance interventions	No eHealth weight loss intervention; information-only interventions; main focus on the treatment of a disease
**Control**	Not applicable	Not applicable
**Outcome**	Weight change (kg or %) from baseline or change in BMI (kg/m^2^)	Weight loss not a primary outcome (main focus, e.g., methodology, theory use, acceptability, or attrition)
**Study design**	Systematic reviews, may include both RCTs and/or NRSIs	Not a systematic review

*BMI* body mass index (calculated as weight in kilograms divided by height in meters squared), *RCT* randomized controlled trial, *NRSI* non-randomized study of intervention

### Search Methods

We searched several databases, including PubMed, Ovid Medline, PsycNet, Scopus, Web of Science, Cochrane Library, Global Index Medicus, and the Centre for Review and Dissemination.

To make the searches as comprehensive as possible, we identified several search term synonyms for population (obesity, obese, overweight) and intervention (telemedicine, telehealth, digital health, eHealth, mHealth, web-based), utilizing and expanding Medical Subject Headings (MeSH) keywords whenever possible. We also combined these search terms with Boolean operators to account for different combinations. While our keywords were in English, we did not limit the search results to any specific language. When possible, we limited the search based on article type (reviews only) and publication date (from January 1st 2018 to February 27th 2023).

We went through the reference lists of included studies to find possible articles not found through our electronic searches. Additionally, we searched the reference lists of non-systematic reviews and umbrella reviews encountered through our primary search. We also searched through several potential databases and registries for gray literature.

A full search strategy, including all searched databases and registries, can be found in the Online Resource.

### Selection Process

Two reviewers (SK and AJ) independently screened all records comparing them to the pre-existing inclusion and exclusion criteria. The selection process was carried out in two phases: first, clearly ineligible records were excluded based on their titles and abstracts. Then, full texts were retrieved and analyzed. If the reviewers did not reach consensus on any given reference, a third reviewer (LS) made the decision.

### Data Extraction

We extracted basic information from the reviews, including aims, inclusion criteria, number of studies included, age, percentage of women, body mass index (BMI; calculated as weight in kilograms divided by height in meters squared), duration, country or ethnicity of participants, outcomes, behavior change technique employed, and funding sources. For reviews with meta-analysis, we extracted key values such as heterogeneity, arms compared, and effect sizes. Two independent reviewers (SK and AJ) extracted the data used in this study.

Several primary studies were included in multiple meta-analyses included in this review, making a meta-analysis of meta-analyses not viable due to issues related to statistical independence [16].

### Methodological Quality Assessment

We evaluated the overall confidence of the results of each review using the AMSTAR 2 tool [18]. It is feasible for evaluating reviews consisting of not only randomized controlled trials (RCTs) but also non-randomized studies of interventions (NRSIs). It consists of 16 items, 13 of which focusing on the review itself and 3 on meta-analysis. The tool is not meant to produce a summary score. Instead, the aim is to evaluate whether the review has one or multiple critical flaws (i.e., suboptimal literature review, risk of bias not discussed adequately, poorly chosen methodology for meta-analysis) or non-critical flaws (i.e., data extraction leaving out important domains, authors not selecting studies or extracting data in duplicate, no list of excluded studies). To score high, the review needs to have no critical weaknesses, and to score moderate, only one critical weakness is accepted. Multiple non-critical weaknesses lower the overall quality of the study.

The developers of AMSTAR 2 encourage that researchers adapt the tool to their specific requirements. For the purpose of this review, we deemed that not including a reference list of excluded studies should not be considered a critical quality flaw. While only a minority of reviews included a reference list of excluded studies, most had stated the number of excluded references for each reason, suggesting that they had documented the reasons for exclusions even if not explicitly reported.

## Results

### Included Reviews

**Fig. 1:**
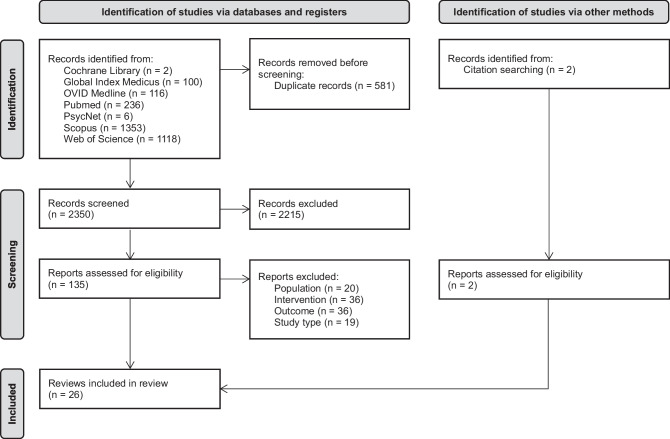
Flowchart of the record screening process

Through our searches, we found 2933 reports in total (Fig. 1). We excluded 581 duplicate reports and 2215 reports not fulfilling our inclusion criteria based on the information provided by their title and abstract. For the remaining 137 reports, we retrieved the full text for further evaluation. After this final evaluation, we included 26 systematic reviews in this review. The exclusion reasons for the other articles retrieved for full text screening can be found in the Online Resource. The 26 included reviews covered a total of 338 original studies (Online Resource).

#### Description of Included Reviews

Tables 2 and 3 describe the characteristics of the included reviews. Funding information can be found in the Online Resource. Fourteen studies focused on eHealth interventions in a general sense [19–32], five on interventions delivered through mobile phones [33••, 34–37], and three on interventions combining eHealth and human-delivered care [38•, 39, 40]. One compared web-based interventions to mobile phone applications [41]. One investigated a variety of behavioral and pharmacotherapy weight loss and weight loss maintenance interventions, 20 of which were technology-based [6]. Three studies focused on weight loss maintenance through eHealth interventions [34, 42, 43].

**Table 2:** Characteristics of the included systematic reviews

Author(s), publication year	Aim of the review	Inclusion criteria of studies	Included studies (*n*)	Duration of the intervention, mean or range	Outcomes	Theoretical/behavioral frameworks/techniques utilized
Ang et al. (2021)	Determine the efficacy of interventions incorporating apps for weight loss and health behavior change in the Asian population	Published in English, interventions delivered partially or fully through a mobile app, minimal study duration 2 mo, age ≥ 18, participants were of Asian ethnicity, outcome weight change	21	Mean 18 wk, range 8 wk to 52 wk	Weight change (kg or %), BMI, waist circumference	N/A
Antoun et al. (2022)	To review studies evaluating the effectiveness of smartphone apps on weight loss when combined with other interventions	RCTs, included the use of a smartphone app, weight loss as an outcome, adults only	34	6 wk to 96 wk	Mean weight change (kg) from baseline to 3, 6, and 12 months	Social cognitive theory, transtheoretical model, self-efficacy theory
Beleigoli et al. (2019)	Investigate the effectiveness of web-based digital health interventions for weight loss and lifestyle habit changes	BMI ≥ 25, age ≥ 18, no pregnant people, RCTs examining web-based digital interventions vs. offline or in-person interventions or waitlist, overweight or obesity as a primary selection criterion	11	N/A	weight, BMI	Several behavioral strategies, e.g., goal setting, self-monitoring and management, social support, modeling and feedback
Berry and Kassavou et al. (2021)	Examine if digital self-monitoring of diet and PA is effective at supporting weight loss, increasing PA or improving eating behavior in adults with obesity or overweight, explore intervention components that might explain variations in effectiveness	BMI ≥ 25, age ≥ 18, interventions enabling digital self-monitoring of both eating behavior and PA, RCTs, comparison groups did not use digital self-monitoring	12	3 mo to 12 mo	Weight loss, physical activity, eating behavior	Self-monitoring, goal setting, feedback, social support, education
Berry and Sala et al. (2021)	Examine the effectiveness of automated digital interventions for improving the outcomes of human coach-delivered weight loss treatment	Individuals aged ≥ 18 with overweight or obesity, peer-reviewed academic journal articles written in English, reported directly quantitative outcomes of an RCT or quasi-RCT with a prepost design, at least one arm with treatment supplemented with a fully automated digital intervention and one without, ADI did not solely track calorie intake	13	12 wk to 24 mo	weight change	Behavior change theory, self-regulation theory, social cognitive theory, health belief model
Besson et al. (2020)	To examine the effectiveness of theoretical and operational inputs in digital interventions for healthy eating	Published in English, adult participants free from other acute illnesses or chronic disease, digital devices primary means of intervention in at least one treatment group, outcome weight loss or weight maintenance	15	mean 32 wk (SD 25.6)	Quantitative measures relating to weight loss or weight loss maintenance	Monitoring, feedback or social support
Chew et al. (2022)	Examine the effects, and the sustainability of these effects, of smartphone apps on anthropometric, metabolic, and dietary outcomes	BMI ≥ 25 for Western populations or ≥ 23 for Asian populations, weight loss as an outcome, reported outcomes beyond the baseline and after the intervention, RCTs, written in English	16	12 wk to 24 mo, FU 8 wk to 24 mo	Weight loss, waist circumference, HDL, LDL, HbA1c, energy intake, blood pressure	Self-monitoring, social support, goal setting, feedback
Dounavi et al. (2019)	To identify existing evidence on the efficacy of mobile health technology in facilitating weight management behaviors	Peer-reviewed primary studies published between 2012 and 2017, adults of typical intellectual ability only, outcome weight loss or management	39 (17 NRSIs, 22 RCTs)	N/A	Weight change, weight management behaviors, use of mobile technology	Feedback, peer support, goal setting, self-monitoring
Holmes et al. (2018)	Examine the effectiveness of interventions using digital technology for weight loss maintenance	Main outcome weight loss maintenance, digital health technologies only, RCTs, published in English between 2006 and February 2018	7	3 mo to 30 mo, mean 12 mo	BMI, BMI-SDS (BMI Standard Deviation Score), weight change	Self-monitoring and reporting, behavior reinforcement
Houser et al. (2019)	To identify different types of technologies used for obesity management and their outcomes	Published in English between 2010 and 2017, peer-reviewed, empirical studies, used some form of digital technology, conducted inside the USA, adults with no cognitive impairments, sample size > 10	23	N/A	Weight change (kg or %), change in BMI, physical activity, dietary intake habits, and time spent in sedentary positions	N/A
Huang et al. (2018)	Evaluate the clinical effectiveness of telemedicine on changes in BMI for people with overweight, obesity, diabetes, or hypertension	Age ≥ 19, RCTs, one arm involved any form of telemedicine, one control group receiving usual care or standard treatment, main outcome BMI, original articles published in English or Chinese	25	9 wk to 2 yr	BMI	N/A
Islam et al. (2020)	Assess the efficacy of mobile phone app interventions for weight loss and increasing physical activity	Written in English, mobile app interventions, study design included control group, outcomes included changes in body weight, BMI, or waist circumference	12	6 wk to 9 mo	Weight loss, BMI, PA	N/A
Jahangiry et al. (2021)	Investigate the effectiveness of web-based interventional programs for weight loss	BMI ≥ 25, age ≥ 18, apparently healthy individuals, RCTs on web-based interventions with a non-web user control group, primary outcome percentage change in body weight	8	12 to 24 wk	weight	Theory of planned behavior, social cognitive theory, behavioral change theory, cognitive behavioral theory
LeBlanc et al. (2018)	Investigate the benefits and harms of behavioral and pharmacotherapy weight loss and weight loss maintenance interventions in adults	Age ≥ 18, BMI ≥ 25 or other suboptimal weight-related measure, weight loss or weight maintenance as a primary outcome, population generalizable to the primary care population, controls received no or minimal intervention or were attention controls	124 (20 utilizing eHealth)	N/A	Health outcomes (mortality, morbidity, depression, health-related quality of life, disability), intermediate outcomes (weight measurements, adiposity measures, incidence or prevalence of obesity-related conditions), adverse events (treatment-related harms)	N/A
Lahtio et al. (2022)	Examine the effectiveness of PA promoting web- and mobile-based distance weight loss interventions in rehabilitation settings on body composition	Age 18–65 years, PA-promoting web- and mobile-based distance weight loss interventions in rehabilitation settings, control groups did not use technology, RCTs, outcomes included BMI, waist circumference, or body fat percentage, published in English, Finnish, or Swedish	30	Mean 30,4 wk, range 4wk to 2yrs	BMI, waist circumference, body fat percentage	N/A
Lau et al. (2020)	Investigate the effectiveness and identify key features of personalized eHealth interventions for weight loss	BMI ≥ 25, age 18–64 years, tailored eHealth interventions incorporating one or more behavioral change techniques, controls received no eHealth intervention, primary outcome weight change	15	12 to 48 wk	Weight	Diverse behavioral strategies, e.g., self-monitoring, goal setting, social support, motivational interviewing and/or prompts
Lee et al. (2022)	To review RCTs on weight loss interventions using digital health for employees with obesity	Adult employees with overweight or obesity, weight loss interventions using digital health, outcome weight or BMI, RCTs published in English or Korean	11	12 wk to 12 mo	Weight change (kg)	Social cognitive theory
Mamalaki et al. (2022)	To examine the effects of technology-based interventions for weight loss maintenance	RCTs, published in English, adults only, at least one web- or app-based intervention arm vs. a control group of minimum intervention or in-person intervention, outcome weight change after the weight maintenance period	12	3 mo to 30 mo	Weight change	Self-monitoring, goal setting, feedback
Mata-Gonzáles et al. (2020)	Examine the efficacy of online interventions for weight loss for adults	Open-access original articles published in English or Spanish, online weight loss interventions, quantitative empirical studies, participants aged 18–60 with overweight or obesity	21	6 wk to 140 wk	Primary outcome change in weight (kg), secondary outcomes body fat, waist circumference, BMI	Self-monitoring, goal setting, social support, feedback
Novaes et al. (2022)	To evaluate the efficacy of digital and hybrid interventions for weight loss for people with severe mental illness	People with severe mental illness (e.g. bipolar disorder, psychotic disorders), illness not in the acute phase, remote or hybrid conducted psychoeducative interventions for weight loss and improved health behavior, age 18–65 years; outcomes related to obesity and weight loss	16 (7 remotes, 9 hybrids)	Remote: 8 wk to 48 wk; hybrid: 10 wk to 52 wk	Change in weight (BMI), outcomes related to metabolic markers and metabolic syndrome	N/A
O’Boyle et al. (2022)	To evaluate the use of mobile technology versus web-based interventions and weight loss outcomes with or without individualized clinician feedback in adults with overweight or obesity	RCTs or controlled cohort studies (with n ≥ 25, dropout rate ≤ 20% or > 20% intent to treat calculated), age ≥ 18, studies not focusing on disease, participants in good health and not pregnant, published in English between January 2010 and January 2020	14	8 wk to 24 mo; eHealth 12 wk to 18 mo	Weight	Self-monitoring, feedback, goal setting
Podina et al. (2018)	Investigate the efficacy of multicomponent behavioral e-health interventions for weight loss	BMI ≥ 25, age ≥ 18, RCTs in which a multicomponent behavioral eHealth intervention was compared with a passive control and/or an active in-person treatment intervention	47	3 mo to 24 mo	Weight loss, BMI, waist circumference, body fat percentage, waist-to-hip ratio, PA, eating behaviors	Social cognitive theory in most interventions; most used techniques were intention formation, self-monitoring, feedback, social support
Puigdomènech et al. (2019)	Examine the efficacy and safety of mHealth interventions (mobile phone apps) for weight control, overweight, and obesity management	assessed the efficacy and/or safety and/or effectiveness of mHealth interventions for overweight or obesity management, sample size over 10	28	3 wk to 24 mo	Efficacy (changes in PA and diet), safety	Feedback, goal setting, self-monitoring
Rumbo-Rodriguez et al. (2020)	Examine how different types of technologies (e.g., mobile phones, internet, social networks, virtual reality) may aid in weight loss in patients with overweight or obesity	BMI ≥ 25, age ≥ 18, published in English or Spanish, studies with at least two groups for comparison where at least one group received an intervention through technology, outcomes included weight	47	N/A	weight loss	Self-monitoring, feedback
Shi et al. (2022)	Examine the effectiveness and components of web-based interventions for overweight or obesity	BMI ≥ 25, age ≥ 18, no pregnant people, RCTs examining a web-based intervention, reported weight change as an outcome, original papers written in English or Japanese	97	2 to 24 mo	Weight change, intervention components	Mostly used behavioral approaches in interventions: self-monitoring, social support, goal setting, information about health consequences
Varela et al. (2021)	To assess the effectiveness of Internet-based behavioral treatments for adults with overweight and obesity, includes network meta-analysis	Age 18–65 years, BMI 25–39.9 kg/m^2^, web-based behavioral weight loss interventions, main outcome weight change (kg), RCTs only	15	Median 18.3 wk, range 12 to 48 wk	Weight change (kg)	N/A

*BMI* body mass index (calculated as weight in kilograms divided by height in meters squared), *RCT* randomized controlled trial, *NRSI* non-randomized study of intervention, *ADI *automated digital intervention*,* *PA* physical activity, *SD* standard deviation, *FU* follow up, *wk* week, *mo* month, *yr* year

**Table 3:** Participant characteristics of the included systematic reviews

Author(s), publication year	N	Age (years)	Women, %	BMI	Country (ethnicity)
Ang et al. (2021)	21 173	≥ 18, mean 45.9 (SD 9.84, range 25.8–60.5)	45.1	27.1 (SD 2.47, range 23.0–30.5)	8 in KR (Korean), 5 in CN (Chinese), 2 in IN (Indian), the USA (Filipino American), SG (multiracial), HK (Chinese), JP (Japanese), 2 in TW (Chinese; Indonesian)
Antoun et al. (2022)	16 to 449, mean 113	≥ 18	N/A	≥ 25	22 in the USA, 3 in AU and NZ, SE, DE, the UK, JP, SG, KR
Beleigoli et al. (2019)	1525	18–65	predominantly women	≥ 25	N/A
Berry and Kassavou et al. (2021)	1190	≥ 18	N/A	≥ 25	7 in the USA, 3 in AU, 1 in DE, 1 in the UK
Berry and Sala et al. (2021)	1471	≥ 18	12 out of 13 trials reported participants’ gender characteristics. Across these trials, women formed a weighted mean percentage of 62.76 (IQR 68.95 to 88.03)	BMI ≥ 25	6 studies included predominantly white people (56–77%), 1 only non-white people, 1 study reported 37.5–72.2% of the population being African American, 5 studies did not report ethnicity or race
Besson et al. (2020)	Mean sample size 142 (SD 110.4)	≥ 18	N/A	≥ 25	11 in the USA, AU, KR, BE, FI
Chew et al. (2022)	2870	mean 22.7–70.1	0–90.7	Mean 27.5–36.2	12 in the USA, UK, AU, JP, CN
Dounavi et al. (2019)	N/A	≥ 18	NRSIs: 16 included men and women, 1 only women	“Increased”; in RCTs ≥ 25	NRSIs: 9 in the USA, 3 in the UK, AU, CA, CN, NZ, N/A; RCTs: 8 in the USA, 5 in AU, 2 in FI, CA, RU, IE, 1 in the NL, 1 in the UK, KR
Holmes et al. (2018)	1939, mean 277, range 34–1032	Mean in children 9.9, mean in adults 44.5	Mean 63	N/A	N/A
Houser et al. (2019)	from 20 to nearly 2500	adults	N/A	N/A	US
Huang et al. (2018)	6253	≥ 18	N/A	N/A	N/A
Islam et al. (2020)	1714	mean 12.7–44.9	mostly women	N/A	5 in AU, 4 in North America, 2 in Europe, 1 in Asia
Jahangiry et al. (2021)	779	≥ 18	N/A	≥ 25	USA, AU, DE, KR
Lahtio et al. (2022)	6103	mean 40.2	Mean 58	N/A	N/A
Lau et al. (2020)	5816	18–64	N/A	≥ 25	USA, AU, IL
LeBlanc et al. (2018)	272 526	Mean 22.7–65.1, range ≥ 18 ≤ 80 years	N/A	N/A	71 in the USA, 28 in Europe (excluding the UK), 16 in the UK, 5 in AU or NZ, 3 in CA, 3 in JP
Lee et al. (2022)	13 392	≥ 18, reported means from 34.2 to 51	N/A	N/A*	4 in the USA, 2 in JP, 2 in DE, DK, IR, the NL
Mamalaki et al. (2022)	2941	≥ 18	N/A	N/A	N/A
Mata-Gonzáles et al. (2020)	9897 (from 49 to 6795)	18–60	N/A	≥ 25	10 in the USA, 7 in AU, 2 in the UK, CA, AT
Novaes et al. (2022)	Remote: 739 (from 15 to 333, mean 105.6); hybrid 1145 (from 13 to 412, mean 127)	Remote: means from 37 to 55.5; hybrid: between 18 and 65	Remote: 45; hybrid: 55.19	N/A	remote: 4 in the USA, CH, TW, ZA; hybrid: 6 in the USA, 2 in the UK, the NL
O’Boyle et al. (2022)	mHealth from 25 to 351, eHealth from 60 to 398	from 18 to 68; eHealth 18 to 75	N/A	≥ 25	N/A
Podina et al. (2018)	15 349	≥ 18	N/A	≥ 25, reported means 29–35.7	N/A
Puigdomènech et al. (2019)	Sample size from 10 to 15 310, 17 studies had a sample size of less than 100	N/A	Majority of women	N/A	17 in the UK, 3 in AU, 2 in KR, UK, BE, ES, the NL, CN, IL
Rumbo-Rodriguez et al. (2020)	22 918	Mean 40.9	51.1	≥ 25	34 in the USA, 3 in AU, 2 in ES, 3 in CN, 2 in the UK, IT, KR, IR
Shi et al. (2022)	N/A	Mean 20.5–69.0	N/A	Mean 26.1–38.1	57 in the USA, 10 in AU, 8 in the UK, 6 in JP, 5 in the NL, 3 in ES, IR, CA, TR, FI, FR, PL, KR, HK
Varela et al. (2021)	2426	≥ 18	48.4	29–33.9	8 in AU, 7 in the USA

*BMI* body mass index (calculated as weight in kilograms divided by height in meters squared), *RCT* randomized controlled trial, *NRSI* non-randomized study of intervention, *SD* standard deviation, *IQR* interquartile range, *USA* United States of America, *UK* United Kingdom, *AU* Australia, *NZ* New Zealand, *CA* Canada, *JP* Japan, *KR* Korea (the Republic of), *CN* China, *IN* India, *SG* Singapore, *HK* Hong Kong, *TW* Taiwan, *SE* Sweden, *DE* Germany, *BE* Belgium, *FI* Finland, *RU* Russia, *IE* Ireland, *NL* Netherlands, *IL* Israel, *DK* Denmark, *AT* Austria, *CH* Switzerland, *ZA* South Africa, *ES* Spain, *IT* Italy, *IR* Iran, *TR* Turkey, *FR* France, *PL* Poland

Fourteen of the studies included only RCTs or quasi-RCTs. Most reviews included a majority of trials concluded in the USA, Australia, or the UK. One review focused on only Asian populations [33••]. Most reviews that included information on the gender characteristics reported that a majority of the participants were women. The duration of the interventions ranged from 3 weeks to 24 months. Social cognitive theory was the most often reported theory framework used. Common characteristics of included interventions were self-monitoring, goal setting, social support, and feedback. The methods for follow-up and outcome measures varied considerably between studies. While some studies incorporated scales utilizing wireless communication, most outcome data were self-reported.

#### Methodological Quality of Included Reviews

We rated two reviews as being of moderate methodological quality [6, 37], 13 as low [19, 20, 23, 25–27, 29, 31, 32, 33••, 34, 38•, 43], and 11 as critically low [21, 22, 24, 28, 30, 35, 36, 39–42]. A detailed evaluation of each AMSTAR 2 criteria can be found in the Online Resource. A considerable number of studies were rated as lower quality because the authors did not justify language restrictions in their search or included studies, did not include a list of excluded studies, or did not justify why they restricted their scope to only some study designs (notably RCTs only). If even one of these issues applicable had been addressed, both studies rated “moderate” and one rated “low” would have been rated “high,” and five of the studies rated “low” would have scored “moderate”. All studies evaluated as being of critically low quality had other substantial flaws and would not have risen in quality appraisal even if the authors addressed these aforementioned issues.

### Efficacy of eHealth Interventions for Weight Loss

The effect sizes of individual meta-analyses are reported in Table 4. It also includes measures of heterogeneity, sample size, and number of studies included in the analyses. All in all, eHealth interventions were concluded to have a positive impact on weight loss results. However, across the reviews, effect sizes range from small to moderate, implying that although statistical significance was achieved, the clinical impact may not be substantial. All reviews highlighted notable heterogeneity among the results of the included studies. The results of interventions delivered via smartphone were similar to those delivered via computer.

**Table 4:** Summary of meta-analyses

Author(s), publication year	Review and comparison	No. of included studies	Outcome (units)	N	Heterogeneity				Effect size (95% CI)	*P*
					χ^2^ (df)	*P*	T^2^	I^2^		
Ang et al. (2021)	Intervention using mobile app vs. control (RCTs only)	14	Weight change (kg)					68.3	− 0.26 (− 0.41, − 0.11) SMD	< 0.01
	Intervention using mobile app vs. control (RCTs only)	11	Change in BMI (kg/m^2^)					69.9	− 0.21 (− 0.42, − 0.01) SMD	0.04
	App combined with usual care vs. usual care (RCTs only)	11	Weight change (kg)					67.6	− 0.28 (− 0.47, − 0.09) SMD	< 0.01
	App combined with usual care vs. usual care (RCTs only)	7	Change in BMI (kg/m^2^)					76.2	− 0.27 (− 0.54, 0.01) SMD	0.05
Antoun et al. (2022)	Smartphone app combined with nonapp intervention vs. control without app, at 3 mo	14	Weight change (kg)	1673	66.71 (13)	< 0.001		81	− 1.99 (− 2.19, − 1.79) MD	< 0.001
	Smartphone app combined with nonapp intervention vs. control without app, at 6 mo	17	Weight change (kg)	2368	161.48 (16)	< 0.001		90	− 2.80 (− 3.03, − 2.56) MD	< 0.001
Beleigoli et al. (2019)	Web-based only vs. offline intervention	10	Weight change (kg)	1497	141.32 (9)	< 0.001	4.43	94	− 0.77 (− 2.16, 0.62) MD	0.28
	Web-based only vs. offline intervention	8	Change in BMI (kg/m^2^)	1244	52.98 (7)	< 0.001	0.46	87	− 0.12 (− 0.64, 0.41) MD	0.66
	Web-based only vs. offline intervention for studies with < 6 months follow-up duration	4	Weight change (kg)	393	2.96 (3)	0.40	0.00	0	− 2.13 (− 2.71, − 1.55) MD	< 0.001
	Web-based only vs. offline intervention for studies with ≥ 6 months follow-up duration	6	Weight change (kg)	1104	79.81 (5)	< 0.001	5.18	94	− 0.17 (− 2.10, 1.76) MD	0.86
	Web-based only vs. active nontechnology intervention	6	Weight change (kg)	1051	9.71 (5)	0.08	0.38	49	0.82 (0.06, 1.59) MD	0.04
	Web-based only vs. wait list	5	Weight change (kg)	886	46.85 (4)	< 0.001		91	− 2.14 (− 2.65, − 1.64) MD	< 0.001
Berry and Kassavou et al. (2021)	Digital intervention vs. control	12	Weight change (kg)	1190	34.96 (11)	< 0.001	1.60	69	− 2.87 (− 3.78, − 1.96) MD	< 0.001
Berry and Sala et al. (2021)	Coaching combined with automated digital intervention vs. offline coaching	13	Weight change (kg)	1471	156.18 (12)	< 0.001	0.50	92	− 0.54 (− 0.95, − 0.13) SMD	0.01
Chew et al. (2022)	Smart phone app intervention vs. control, < 3 mo	8	Weight change (kg)	1021		< 0.01	4.63	91.3	− 1.15 (− 3.02, 0.72) MD	0.19
	Smart phone app intervention vs. control, 3 mo	11	Weight change (kg)	1406		< 0.01	3.80	87.3	− 2.18 (− 3.59, − 0.78) MD	< 0.01
	Smart phone app intervention vs. control, 6 mo	13	Weight change (kg)	1604		0.01	2.02	52.4	− 2.15 (− 3.25, − 1.05) MD	< 0.01
	Smart phone app intervention vs. control, 9–12 mo	5	Weight change (kg)	899		0.52	0.03	0	− 1.63 (− 2.99, − 0.26) MD	0.03
Huang et al. (2018)	Telehealth weight loss intervention vs. control	5	Change in BMI (kg/m^2^)			0.17		37.2	− 0.68 (− 1.07, − 0.30) MD	< 0.01
Islam et al. (2020)	Mobile phone app intervention vs. control	11	Weight change (kg)		Q = 42.65	< 0.1	1.40	76.6	− 1.07 (− 1.92, − 0.21) MD	0.01
	Mobile phone app intervention vs. control	10	Change in BMI (kg/m^2^)		Q = 42.65	< 0.1	0.17	78.0	− 0.45 (− 0.78, − 0.12) MD	0.008
	Mobile phone app intervention vs. control, ≤ 3 mo	5	Weight change (kg)						− 0.004 (− 0.79, 0.80) MD	0.99
	Mobile phone app intervention vs. control, > 3 mo	6	Weight change (kg)						− 1.63 (− 2.64, − 0.61) MD	< 0.002
Jahangiry et al. (2021)	Web-based intervention vs. non-web-based control	8	Weight change (kg)	779		0.001		74.8	0.56 (− 3.47, 4.59) WMD	0.786
Lahtio et al. (2022)	Web- or mobile-based distance weight loss intervention vs. no technology control	28	Change in BMI (kg/m^2^)	4060		< 0.001		90	− 0.83 (− 1.15, − 0.51) MD	< 0.001
Lau et al. (2020)	Personalized eHealth intervention vs. control, population with obesity	12	Weight change (kg)	1640					− 3.10 (− 4.05, − 2.15) MD	< 0.001
	Personalized eHealth intervention vs. control before sensitivity analysis	11	Change in BMI (kg/m^2^)	5317				80	− 0.77 (− 1.03, − 0.51) MD	< 0.001
	Personalized eHealth intervention vs. control after sensitivity analysis	9	Change in BMI (kg/m^2^)	1226				13	− 0.92 (− 1.10, − 0,74) MD	< 0.001
Mamalaki et al. (2022)	Technology-based weight loss maintenance intervention vs. minimum intervention	10	Weight change (kg)	2187	Q = 4.63 (df 9)	0.87	0.00	0.0	− 0.07 (− 0.57, 0.42) MD	0.77
	Technology-based weight loss maintenance intervention vs. in-person intervention	4	Weight change (kg)	1105	Q = 4.78 (df 3)	0.19	0.45	37.2	1.36 (0.29, 2.43) MD	0.01
Podina et al. (2018)	eHealth intervention vs. passive control at posttreatment	50	Weight outcomes					84	0.34 (0.24, 0.44) g	
	eHealth intervention vs. passive control at follow-up	10	Weight outcomes					84	0.25 (0.05, 0.46) g	
	eHealth intervention vs. active control at posttreatment	7	Weight outcomes					< 0.01	− 0.31 (− 0.43, − 0.20) g	
Shi et al. (2022)	Web-based intervention vs. offline control	44	Weight change (kg)	7062	481.60 (43)	< 0.001	0.29	91	− 0.57 (− 0.75, − 0.40) SMD	< 0.001
Varela et al. (2021)	Intensive contact website^a^ vs. wait list	3	Weight change (kg)	320	0.94 (2)	0.63	0.00	0	− 4.32 (− 5.08, − 3.57) MD	< 0.001
	Minimal contact website^a^ vs. wait list	3	Weight change (kg)	480	0.81 (2)	0.67	0.00	0	− 3.23 (− 3.80, − 2.66) MD	< 0.001
	Intensive contact website vs. self-help	5	Weight change (kg)	790	0.40 (4)	0.98	0.00	0	− 1.79 (− 2.33, − 1.24) MD	< 0.001

*BMI* body mass index (calculated as weight in kilograms divided by height in meters squared), *RCT* randomized controlled trial, *MD* mean difference, *SMD* standardized mean difference, *WMD* weighted mean difference, *g* Hedge’s g, *Q* Cochran’s Q, *I*^2^ percentage of the variation across studies that can be attributed to study heterogeneity, *T*^2^ between-study variance

^a^Intensive contact website: personalized feedback provided by a healthcare professional at least once a week. Minimal contact website: feedback provided at least once a month; provided by a professional (personalized) or machine (not personalized)

The results of narrative reviews are described in Table 5. Overall, they concluded that eHealth interventions are associated with weight loss outcomes that are at least comparable, and in some cases superior, to those achieved through control conditions.

**Table 5:** Summary of narrative reviews

Author(s), publication year	Number of studies	Main findings
Besson et al. (2020)	15	Digital interventions can be beneficial in weight loss for adults with overweight. Nine studies out of fifteen showed a significant difference between treatments favoring digital interventions. Also, the results of the rest of the studies (6) indicated that when comparing the digital interventions to the active compare group, they could be as successful as the comparison intervention. Interventions that promoted weight loss utilized, e.g., personalized feedback and counseling, social support, and self-monitoring through web programs, Internet chats, text messages, and mobile apps. The results were mixed when digital interventions were compared to traditional face-to-face/human counseling interventions: some found that hybrid (human and digital) intervention was the most effective for weight loss, whereas some did not find the difference. Digital interventions succeeded well in the short term compared to human intervention, but human counseling was more effective in the longer term
Dounavi et al. (2019)	39	The results of both RCTs and non-randomized studies showed that digital apps were useful and easy to use for the purposes of weight management and weight loss. Including tools that enabled the effortless self-monitoring of health behaviors, interaction with peers, tailored feedback, and reminders to continue with the app, increased engagement in the process and thus supported successful weight management
Holmes et al. (2018)	7	The use of digital health technologies promoted successful weight-loss maintenance in short-term periods (3–24 months). Four RCTs out of seven reported that compared to controls with no contact or face-to-face contact; the technology significantly aided the weight management process. Self-monitoring and reporting were essential components of digital health technologies. The results of five trials suggested that digital interventions could support goal setting and social interaction. Also, personalized contacts were seen as necessary for participants
Houser et al. (2019)	23	The statistically significant association between the use of digital components and weight loss was seen in 14 studies. The review noticed that 73% of the studies that utilized mobile health devices showed a statistically significant association between weight loss and used technology, whereas 40% of the studies that used telemedicine and 50% of the studies that used eHealth reported statistically significant differences. Digital tools were utilized to provide reminders and encourage health-promoting behaviors
Lee et al. (2022)	11	All studies showed a statistically significant weight loss after the digital health intervention. Seven studies reported a significant difference between the intervention and comparison groups, and among those studies, six showed that both intervention and control groups lost weight. Still, the intervention groups’ weight reduction was greater than the control groups. The remaining four studies failed to show a significant difference between the groups. Intervention strategies included in the studies were, e.g., tailored advice, personalized goal settings and daily messages, tailored remote group meetings, and online social support
Mata-Gonzáles et al. (2020)	21	The online intervention showed significant differences in weight when compared to the control group or face-to-face intervention. Web-based programs that promoted weight management focused on, e.g., increasing physical activity and making healthier changes to diet. Successful interventions utilized self-monitoring and social support as well as goal setting
Novaes et al. (2022)	16	Both remote and hybrid (digital + face-to-face) interventions found significant outcomes favoring the interventions. However, as statistical methods and study outcomes varied, direct comparisons were difficult to make. In conclusion, digital approaches seem practical, competent, and valuable tools to reduce sedentary behavior, improve life quality and healthier lifestyle, and lose weight among patients with severe mental illnesses
O’Boyle et al. (2022)	14	The review found that both mHealth (8) and eHealth (6) interventions positively impacted weight loss and behavior change. When combining human support (regular clinician coaching through, e.g., phone calls, text messages, and e-feedback) with digital components, the participants gained the most successful outcomes regarding weight and behavior change. Self-regulation, reporting weight-related behaviors, and tailored feedback were essential factors in successful interventions
Puigdomènech et al. (2019)	28	The review aimed to find methods of how mHealth interventions had evaluated the efficacy, effectiveness, and safety of digital interventions for weight loss/management. Most of the studies (78%) assessed the reduction in weight/BMI as a primary marker for the efficacy of mHealth intervention, followed by changes in physical activity and diet. Feedback messaging, goal setting, and self-monitoring were the most used tools in apps. Peer support and gamification might be useful to increase engagement and motivation and thus improve the efficacy of the intervention. The weight loss results were controversial: some found no difference between intervention and control groups, whereas some found significant or non-significant reductions in weight-loss markers between groups. Interventions that included face-to-face elements in their program obtained the most successful outcomes
Rumbo-Rodriguez et al. (2020)		Different technologies such as smartphones, apps, websites, and personal digital assistants were used in weight loss interventions for patients with overweight and obesity. Almost half of the interventions (47%) reported a significant impact of the technology-based interventions for weight loss compared to the control or comparison group. The use of digital tools also seems to improve treatment adherence as they offer more straightforward and faster self-monitoring via technology. Also, some findings indicate that the adherence level is further increased when it is accompanied by immediate feedback. Additionally, the short-term weight loss results highlight the crucial role of personalized feedback in weight management, but the association has not been observed in the long term

#### eHealth Interventions vs. Any Control Conditions

Seventeen reviews reported weight loss outcomes by comparing intervention effects to any control conditions, including standard care, waitlists, paper-based communication, or minimal interventions such as an educational website or a single face-to-face educational session [19, 20, 22–28, 30, 31, 34–37, 40, 42].

The results favored eHealth interventions, with mean differences of statistically significant (*P* < 0.05) weight loss outcomes ranging from − 1.07 (95% CI − 1.92, − 0.21) kg to − 3.10 (− 4.05, − 2.15) kg for absolute weight change and from − 0.12 (− 0.64, 0.41) kg/m^2^ to − 0.92 (− 1.10, − 0.74) kg/m^2^ for changes in BMI. Of the 15 reviews that included meta-analysis, only two did not find significant differences between weight loss outcomes [19, 24]. All 8 narrative reviews concluded that eHealth interventions reach at least similar and often even greater weight loss results than control conditions.

#### eHealth Interventions vs. No or Minimal Interventions

Three reviews compared eHealth interventions to minimal interventions, finding the former to be more effective. The control conditions varied, but they included no interventions (e.g., waitlist or no care) or minimal interventions (e.g., newsletter or information pamphlet). Podina et al. found effect sizes to be small but consistent across trials (Hedge’s g 0.34 (0.24, 0.44)) [29]. Beleigoli et al. found those in the eHealth intervention to achieve better weight loss results than those on a wait-list (mean difference (MD) − 2.14 (− 2.65, − 1.64) kg, *P* < 0.001) [19]. Moreover, Varela et al. reported that those in an eHealth intervention with professional feedback at least weekly reached the strongest outcomes compared to those on a wait-list (MD − 4.32 (− 5.08, − 3.57) kg, *P* < 0.001) while those in a mostly automated eHealth intervention reached slightly weaker results (MD − 3.23 (− 3.80, − 2.66) kg, *P* < 0.001) [32].

#### eHealth Interventions Combined with Professional Contact

All three reviews examining the effects of feedback found that eHealth interventions with personalized feedback provided by a health professional were significantly more effective than interventions with no or machine-generated feedback, with mean differences in weight loss outcomes as great as − 4.3 kg (95% CI − 5.08, − 3.57 kg) [25, 26, 32]. Similarly, Varela et al. concluded that web-based interventions with professional contact were more effective than self-help websites (MD − 1.79 (− 2.33, − 1.24) kg) [32].

Both in-person and e-counseling were found to increase intervention success. Puigdomènech et al. concluded that interventions including face-to-face elements obtained the most successful outcomes [37], while Shi et al. found that eHealth interventions with e-counseling had better results than those without (standardized mean difference (SMD) − 0.42 (− 0.75, − 0.08) kg, *P* = 0.04) [31]. This finding was supported by O’Boyle et al., who found that phone calls, text messages, and e-feedback also enhance intervention success [41]. Besson et al. stated mixed results, where some studies found hybrid interventions to be more effective than eHealth interventions only, while others did not find a difference [21].

Combining face-to-face interventions with an eHealth intervention also leads to enhanced weight loss results. Berry and Sala et al. found that combining human coaching with an automated digital intervention provided better results than coaching alone (MD − 2.18 (− 4.39 kg to − 0.03 kg) and − 2.21% (− 4.49 to 0.08%) in body weight from baseline) [39]. Similarly, both Ang et al. and Antoun et al. found that combining usual care with a mobile application was more effective than usual care alone (Hedge’s g − 0.28 (− 0.47, − 0.09); *P* < 0.01 and MD − 2.80 (− 3.03, -2.56) kg; *P* < 0.001, respectively) [33••, 38•]. Surprisingly, Berry and Sala et al. concluded that combined interventions with lower duration or frequency of coach contact (< 10 h) provided better results than interventions with more intensive coaching (> 10 h) (SMD 0.11 (− 0.24, 0.45) kg vs. 0.86 (0.20, 1.52) kg, *P* = 0.05).

#### eHealth Interventions vs. Face-to-Face Interventions

Four reviews reported weight loss outcomes in eHealth interventions compared with face-to-face interventions, such as usual care without an eHealth component. Beleigoli et al. found that stand-alone web-based interventions had poorer weight loss outcomes than in-person interventions (MD 0.82 (0.06, 1.59) kg, *P* = 0.04) [19]. Similarly, Podina et al. found active control groups to have more beneficial results than eHealth groups (Hedge’s g − 0.31 (− 0.43, − 0.20)). On the other hand, Besson et al. found that eHealth interventions could be as successful as active offline comparison interventions or even more effective in the short term [21].

### Efficacy of eHealth Interventions for Weight Loss Maintenance

The outcomes of eHealth interventions on weight loss maintenance seem less pronounced than on initial weight loss. Mamalaki et al. found that eHealth interventions produced similar results than minimal interventions while leading to greater weight regain in comparison with in-person care [43]. In contrast, Podina et al. found eHealth interventions to be more effective in weight loss maintenance than passive control conditions, although the effect sizes remained small (Hedge’s g = 0.25 (0.05, 0.46) before and 0.15 (0.02, 0.27) after sensitivity analysis) [29].

Chew et al. found that only 6% (1 out of 16 studies) of included mobile application interventions reported significant weight loss at both 18- and 24-month follow-up [34]. On the other hand, Holmes et al. concluded that digital health technologies aided in weight loss maintenance in short time periods (3–24 months), with four out of seven RCTs reporting significant improvements compared to no contact or in-person care [42].

## Discussion

This review article summarizes 26 systematic reviews, covering a total of 338 original studies, that evaluate the efficacy of web-based interventions for weight loss or weight loss maintenance. The review indicates that eHealth interventions are more effective than control interventions or no care and comparable to face-to-face interventions. The effect sizes remain relatively small when comparing eHealth interventions to any control conditions, with mean differences of weight loss results from − 0.12 kg (95% CI − 0.64 to 0.41 kg) in a review comparing eHealth interventions to face-to-face care to − 4.32 kg (− 5.08 kg to − 3.57 kg) in a review comparing eHealth interventions to no care. The methodological quality of the included studies varies considerably. However, it can be concluded that interventions with human contact work better than those that are fully automated.

### Features Enhancing Intervention Effect

Features linking effective interventions together were individual feedback, tailored content, self-monitoring, and the use of multiple intervention modalities. A common finding was that interventions with human contact performed better than fully automated interventions. The contact does not have to be time-consuming: Varela et al. found that weekly tailored feedback provided by a professional resulted in more favorable outcomes than no or fully automated feedback [32]. Furthermore, Berry et al. found that when combined with a fully automated intervention, a lighter amount of additional coaching led to greater weight loss results than more intensive coaching [39]. Additionally, personal support has been shown to increase adherence to eHealth interventions [44•, 45–47].

### The Importance of Cultural and Social Awareness

In cultures that value community and interpersonal relationships, close contact with service providers may lead to more favorable outcomes and greater acceptance of interventions. For instance, Ang et al. [33••] discovered that mobile applications developed for Asian populations were typically culturally adapted and commonly enabled patient-professional communication, whereas applications designed for Western populations often relied on self-directed learning. The authors also emphasized the significance of proper localization, including culturally appropriate advice, locally adapted educational content, and the use of native language. Similarly, Rosenbaum et al. noted that racial minorities were more likely to enroll in trials that used both smartphones and in-person care, suggesting that these populations were seeking social support or a sense of connection to service providers [48].

Cultural and social awareness may also improve the feasibility of eHealth interventions when treating vulnerable groups such as immigrants, aging populations, people with low health literacy, or people with low socioeconomic status [4, 44•, 49, 50••, 51]. It is critical to successfully engage these populations in eHealth interventions because these may provide certain advantages over non-eHealth interventions. For example, because eHealth interventions are multimodal, they can deliver information not only in text but also in video or audio format. Moreover, they may alleviate the burden of limited transportation or caregiving duties [48]. eHealth can also enhance healthcare accessibility for previously underserved populations [52].

### From Statistical Significance to Clinical Significance

The majority of trials included in the present reviews reported absolute change in weight (kg) or BMI (kg/m^2^). While results based on these measures may achieve statistical significance, their clinical significance remains uncertain, particularly given inconsistent reporting of participants’ weight at baseline. We need to know what to compare the results to in order to make meaningful assumptions about the clinical significance based on absolute weight change. For example, a 2 kg weight loss in a person who weighs 70 kg at baseline may be significant, but it is not in a person who weighs 120 kg at baseline.

One way to support the evaluation of clinical significance is to report relative weight change (Δ% from baseline). Although this approach is not without limitations, it does produce more useful results. A 5% weight loss is generally considered clinically significant, and even a 3% weight loss is likely to result in positive health changes [53]. Reporting relative weight change also mitigates distortion of final results caused by participants with widely different baseline weights. It acknowledges that people with higher baseline weights require greater absolute weight loss to achieve clinically significant relative weight loss.

We must also keep in mind that reported effect sizes from meta-analyses only show differences between groups, not how patients’ weight changed from baseline. For example, a mean difference only reflects the difference in means between groups, not the difference in means within groups from baseline. Similarly, when reporting standardized mean differences, the assumption should be that the differences in standard deviations are due to different measurement scales rather than real differences and high variability within study populations. Trials may include participants with a broader range of baseline characteristics, leading to higher standard deviation. Given that the included studies in this review exhibited high heterogeneity, it is reasonable to assume that at least some of the differences in standard deviation were due to this variability. Therefore, effect sizes alone do not provide a reliable estimate of the clinical impact of the interventions.

### We Need More Research in Real-Life Settings

While randomized controlled trials are often regarded as the gold standard for intervention research, including non-randomized studies on intervention effects may help us understand how these interventions work in clinical practice. They may be more feasible when addressing long-term outcomes, outcomes in different populations or settings, or alternative methods of intervention delivery. We also know that retention rates may differ between trial and real-life interventions [54]. Subsequently, efficacy information based solely on RCTs conducted in non-real-world settings may not be widely applicable or generalizable when transitioning from trials to healthcare.

Several trials excluded participants with chronic diseases or underlying diagnoses. In reality, many patients with obesity also have concomitant or comorbid diseases such as hypertension, asthma, type 2 diabetes, or heart disease. A recent real-life cohort study by Kupila et al. with nearly 1300 patients enrolled in a digital obesity management program demonstrated that weight loss success was not affected by the number of concurrent diagnoses or medications [55•]. Enrolling a wider selection of patients would likely result in larger trial populations and more clinically generalizable data without introducing significant confounding factors.

Furthermore, we require more long-term research to ascertain the effects of eHealth interventions in managing obesity. Several studies included in the reviews had brief study and follow-up periods. Although these study designs may demonstrate if an eHealth intervention initially works, obesity care and weight management demand long-term and sustainable solutions that integrate into patients’ daily lives. Short-term study durations fail to provide information on how these interventions function in the long run or if their effects last.

### We Need a Change from Theory to Practice

Examining a large body of evidence invites us to consider its practical implications. Many trials employ eHealth interventions created specifically for research purposes. While this approach provides valuable information about eHealth interventions themselves, it does not facilitate the much-needed step towards digitalization, unless interventions are developed for use in clinical practice.

The question “does it work?” has been extensively researched, but there is limited knowledge on “how does it work?”. Simply observing impact or efficacy does not help us develop more effective interventions. Many reviews explore and discuss various aspects of successful interventions. While this information is welcome, we need more trials examining underlying factors that influence intervention success. Weight loss outcomes may vary based on intervention details such as theoretical frameworks used, components, amount of human contact, or modes of delivery. They may also vary due to population differences such as age, socioeconomic status, gender, or baseline health status. We need to know what works and with whom in order to not only develop interventions but also deliver them to those who will most likely benefit from them. On the other hand, we must be able to identify those who may need more face-to-face support.

There is relatively little research on intervention acceptability. Not only is an effective intervention necessary, but users must also accept it as part of their care. Reviews have highlighted that eHealth interventions may work, but only if the users adhere. Developing interventions from top to bottom, from researchers and developers to end-users, may overlook any hidden needs or wishes of patients or professionals. This raises critical unanswered questions, such as which types of interventions patients and professionals believe will benefit them the most, what types of feedback or human contact will best support lifestyle change, and what will help users commit to the intervention and empower them to take agency over their own health and care?

### Risk of Bias in the Included Studies

The majority of the included reviews excluded studies published in languages other than English. Furthermore, the vast majority of studies were conducted in the USA or Europe, which limits the generalizability of their findings to other populations. Although we searched the Global Index Medicus for potential sources published in low- to middle-income countries, we found none that met our inclusion criteria. Nevertheless, it is important to note that we identified thousands of original articles published in languages other than English when we searched with the same keywords but with no restrictions on publication type.

This suggests that existing systematic reviews may overlook a significant number of available publications by restricting their search language. Consequently, this may introduce language and publication bias and skew our understanding of the impact of eHealth innovations by only considering their impact in Western populations.

Most authors provided the number of full-text articles that were excluded and their reasons for doing so. However, only a few authors provided a list of articles excluded after full-text examination. This lack of transparency reduces the credibility of research findings. Therefore, we suggest that authors include a list of excluded articles in future reviews.

We know that a disproportionate number of weight loss intervention participants drop out during the intervention [56]. We did not examine the attrition rates of included reviews as this was not the purpose of this review. However, for an intervention to be effective, its participants must adhere to it. Weight loss results from only those who completed an intervention may be reported in the absence of an intent-to-treat analysis. When applied to a real-world situation, the outcomes will likely differ. Thus, if attrition rates were reported in the original trials, it would be beneficial to report them in future reviews. This would aid the reader in reaching a deeper understanding of the interventions in question.

Although most reviews with meta-analysis observed no significant publication bias, often concluded by visually inspecting forest plots, only a few reviews in this study compared eHealth research interventions to real-life interventions. This raises the question of whether this is due to a lack of study designs that allow for such comparisons or whether it is an indication of publication bias due to potentially unfavorable results. Furthermore, many reviews limited their search language to English or did not search for gray literature (research produced by organizations outside of the traditional commercial or academic publishing), potentially increasing publication bias. While it could be argued that these restrictions would not have affected the final results in a meaningful way, we have no way of knowing what the true impact would have been in the context of any individual review.

### Heterogeneity in the Included Studies

The included reviews generally exhibited a high degree of heterogeneity. The terms “eHealth” and “digital health” lack clear definitions, leading authors to interpret them in their own ways. A single review could have included a variety of intervention modalities, such as phone or video calls, websites, DVDs, rapid messaging, mobile phone applications, or social media. Furthermore, the duration and intensity of the interventions varied, and the participants ranged in age, background, and health status.

With a more focused research question, the included interventions could be narrowed down, resulting in a more uniform review synthesis. This could also be accomplished with adequate subgroup analysis. Combining various interventions in meta-analyses produces non-generalizable results that cannot be interpreted in any specific context. This makes it difficult for clinicians and policymakers to make meaningful recommendations based on available research.

The interventions examined in these reviews were based on a variety of behavior change theories (e.g., the Social Cognitive Theory, the Self-Determination Theory, the Theory of Planned Behavior). It is plausible that frameworks that are effective in real-life settings are also applicable in virtual settings. However, as with most therapeutic interventions, the therapeutic alliance may be a more significant determinant of treatment outcomes than any particular theoretical model [57, 58].

### Strengths and Limitations

We only included systematic reviews in this review because they follow a robust and reproducible methodology that aims to find all relevant trials available. Additionally, we screened the reference lists of other reviews of reviews, both systematic and non-systematic, to identify any additional reviews that could be included. With the large number of systematic reviews found, and our extensive search of gray literature, we are confident that our scope is sufficiently broad and comprehensive.

To ensure the reliability of our review, we followed a predetermined protocol and conducted both reference searches and data extraction in duplicate. We also aimed to report our review process and findings as transparently as possible, with the aid of the additional material included in the Appendix.

We included reviews published in 2018 or later. Although the majority of the trials included in these reviews were published relatively recently, a few were published as early as 2001. This could have partially influenced the results because we know that newer eHealth interventions, specifically those developed in the 2010s or later, yield better results than older interventions [31]. This could be attributed to the rise of digitalization, which has resulted in improved technology literacy and advancements in device and application design.

As previously discussed, the included reviews generally had a high or moderate risk of bias, as well as high heterogeneity among the included trials and results. This must be taken into consideration when interpreting the findings of this study.

## Conclusion

The review indicates that eHealth interventions are more effective than control interventions or no care and comparable to face-to-face interventions. Notably, the most significant weight loss results have been observed in eHealth interventions that combine a digital program with personal counseling or coaching from a qualified professional, delivered either remotely or in-person. Common features of effective interventions include individual feedback, personalized content, self-monitoring, and the utilization of various intervention modalities. However, studies focusing on the maintenance of weight loss are limited, leaving a gap in knowledge regarding the long-term effectiveness of eHealth interventions for weight loss and weight loss maintenance.

### Supplementary Information

Below is the link to the electronic supplementary material.

Supplementary file1 (PDF 1534 KB)
