# Correction: The Effectiveness of eHealth Interventions for Weight Loss and Weight Loss Maintenance in Adults with Overweight or Obesity: A Systematic Review of Systematic Reviews

**DOI:** 10.1007/s13679-023-00530-3

**Published:** 2023-10-14

**Authors:** Sakris K. E. Kupila, Anu Joki, Laura-U. Suojanen, Kirsi H. Pietiläinen

**Affiliations:** 1 Obesity Research Unit, Research Program for Clinical and Molecular Metabolism, Faculty of Medicine, University of Helsinki, Helsinki, Finland; 2 HealthyWeightHub, Endocrinology, Abdominal Center, Helsinki University Hospital and University of Helsinki, Helsinki, Finland

**Correction to: Current Obesity Reports (2023) 12:371-394** 10.1007/s13679-023-00515-2

The original version of this article unfortunately contained errors in Fig. 1 and in the first paragraph of “Included Reviews”.

The authors misquoted the values of reports and hereby publish the correct paragraph and Fig. 1.

## Included Reviews

Through our searches, we found 2933 reports in total (Fig. 1). We excluded 581 duplicate reports and 2215 reports not fulfilling our inclusion criteria based on the information provided by their title and abstract. For the remaining 137 reports, we retrieved the full text for further evaluation. After this final evaluation, we included 26 systematic reviews in this review. The exclusion reasons for the other articles retrieved for full text screening can be found in the Online Resource. The 26 included reviews covered a total of 338 original studies (Online Resource).

**Fig. 1:**
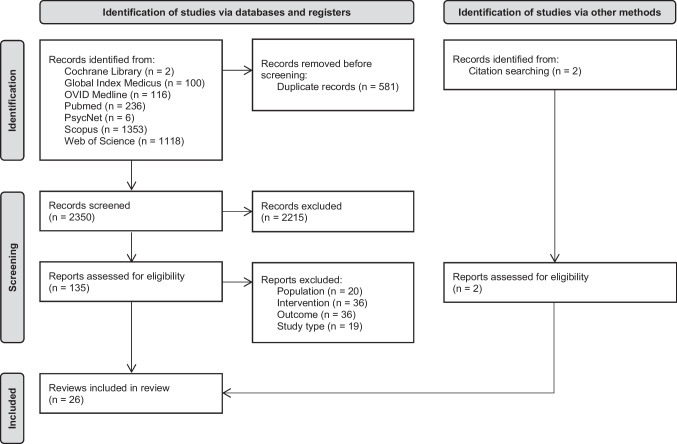
Flowchart of the record screening process

The original article has been corrected.

