# Supplementary material for: The Effectiveness of eHealth Interventions for Weight Loss and Weight Loss Maintenance in Adults with Overweight or Obesity: A Systematic Review of Systematic Reviews
Source: Curr Obes Rep. 2023 Jun 24;12(3):371–94. doi: 10.1007/s13679-023-00515-2 (PMC10482795; doi:10.1007/s13679-023-00515-2)
Supplement: Supplementary file 1 — Supplementary file1 (PDF 1534 KB) [file 13679_2023_515_MOESM1_ESM.pdf]

The effectiveness of eHealth interventions for weight loss and weight loss maintenance in adults with overweight or obesity: a systematic review of systematic reviews

Kupila SK[1], Joki A[2], Suojanen LU[2], Pietiläinen KH[1, 2]

[1] Obesity Research Unit, Research Program for Clinical and Molecular Metabolism, Faculty of Medicine, University of Helsinki, Helsinki, Finland

[2] HealthyWeightHub, Endocrinology, Abdominal Center, Helsinki University Hospital and University of Helsinki, Helsinki, Finland

Contents

Search strategy 2
Additional sources 4
Grey literature 5
Table S1. List of records excluded after full text review 8
Table S2. Studies included in each review 26
Table S3. Funding information of the included reviews. 42
Table S4. Quality of the included reviews 44

Search strategy

Pubmed:

Keywords: ((telemedicine) OR (telehealth) OR (digital health) OR (ehealth) OR (e-health) OR (mhealth) OR (m-health) OR (web-based) OR (web based)) AND ((obesity) OR (overweight) OR (weight loss) OR (weight maintenance) OR (weight management))

filters: Review, Systematic Review, Meta-Analysis, in the last 5 years, Adult: 19+ years

Scopus:

Keywords: ((telemedicine) OR (telehealth) OR (digital health) OR (ehealth) OR (e-health) OR (mhealth) OR (m-health) OR (web-based) OR (web based)) AND ((obesity) OR (overweight) OR (weight loss) OR (weight maintenance) OR (weight management))

limit to medicine, psychology; child, adolescent; document type review; publication year > 2017 and < 2024

Ovid:

Keywords: ((telemedicine) OR (telehealth) OR (digital health) OR (ehealth) OR (e-health) OR (mhealth) OR (m-health) OR (web-based) OR (web based)) AND ((obesity) OR (overweight) OR (weight loss) OR (weight maintenance) OR (weight management))

include related terms; limit to ("review articles" and humans and yr="2018 -Current" and "all adult (19 plus years)"

Web of Science:

SK combined two queries:

#1 ((telemedicine) OR (telehealth) OR (digital health) OR (ehealth) OR (e-health) OR (mhealth) OR (m-health) OR (web-based) OR (web based)) AND ((obesity) OR (overweight) OR (weight loss) OR (weight maintenance)) (All Fields) and Review Article (Document Types)

AND

#2 ALL=(child OR adolescent OR postpartum OR pregnant)

AFTER WHICH

#1 NOT #2 limited to review articles; custom range: 2018-01-01 to 2023-this-day

AJ formatted one query:

(ALL=(((telemedicine) OR (telehealth) OR (digital health) OR (ehealth) OR (e-health) OR (mhealth) OR (m-health) OR (web-based) OR (web based)) AND ((obesity) OR (overweight) OR (weight loss) OR (weight maintenance) OR (weight management)))) NOT ALL=((child OR adolescent OR postpartum OR pregnant OR teen OR teenager OR (bariatric) surgery)) and Review Article (Document Types) and 2023 or 2022 or 2021 or 2020 or 2019 or 2018 (Publication Years)

SK retrieved 725 articles and the AJ 745 articles, after duplicate deletion the final number of retrieved articles was 1118.

APA PsycNet:

Keywords: ((Any Field: telemedicine) OR (Any Field: telehealth) OR (Any Field: digital health) OR (Any Field: ehealth) OR (Any Field: e-health) OR (Any Field: mhealth) OR (Any Field: m-health) OR (Any Field: web-based) OR (Any Field: web based)) AND ((Any Field: obesity) OR (Any Field: overweight) OR (Any Field: weight loss) OR (Any Field: weight maintenance) OR (Any Field: weight management)) AND Any Field: review AND Age Group: Adulthood (18 yrs & older)

limit to adulthood

Cochrane:

https://www.cochranelibrary.com/advanced-search/search-manager

combine two queries:

#1 ((telemedicine) OR (telehealth) OR (digital health) OR (ehealth) OR (e-health) OR (mhealth) OR (m-health) OR (web-based) OR (web based)) AND ((obesity) OR (overweight) OR (weight loss) OR (weight maintenance))

#2 child OR adolescent OR pediatric OR pregnant OR postpartum

#1 NOT #2

limit topics to endocrine & metabolic and further to obesity & overweight

Centre for Review and Dissemination (CRD):

https://www.crd.york.ac.uk/CRDWeb/

Keywords: ((telemedicine) OR (telehealth) OR (digital health) OR (ehealth) OR (e-health) OR (mhealth) OR (m-health) OR (web-based) OR (web based)) AND ((obesity) OR (overweight) OR (weight loss) OR (weight maintenance) OR (weight management)) AND (review)

limit to publication years 2018-2023

Additional sources

REFERENCE LISTS OF PUBLISHED REVIEWS

To collect this list of potential sources of references, we listed reviews (of reviews) encountered during the main screening process as well as professional recommendations made by the authors of this systematic review.

Ryan K, Dockray S, Linehan C. A systematic review of tailored eHealth interventions for weight loss. Digit Health. 2019 Feb 5;5:2055207619826685. doi: 10.1177/2055207619826685. PMID: 30783535; PMCID: PMC6366004.	no new references
Hinchliffe N, Capehorn MS, Bewick M, Feenie J. The Potential Role of Digital Health in Obesity Care. Adv Ther. 2022 Oct;39(10):4397-4412. doi: 10.1007/s12325-022-02265-4. Epub 2022 Aug 4. PMID: 35925469; PMCID: PMC9362065.	one new reference: Dounavi et al. 2019
Wang Y, Min J, Khuri J, Xue H, Xie B, A Kaminsky L, J Cheskin L. Effectiveness of Mobile Health Interventions on Diabetes and Obesity Treatment and Management: Systematic Review of Systematic Reviews. JMIR Mhealth Uhealth. 2020 Apr 28;8(4):e15400. doi: 10.2196/15400. PMID: 32343253; PMCID: PMC7218595.	no new references

Marcolino MS, Oliveira JAQ, D'Agostino M, Ribeiro AL, Alkmim MBM, Novillo-Ortiz D. The Impact of mHealth Interventions: Systematic Review of Systematic Reviews. JMIR Mhealth Uhealth. 2018 Jan 17;6(1):e23. doi: 10.2196/mhealth.8873. PMID: 29343463; PMCID: PMC5792697.	no new references
Kahan S, Look M, Fitch A. The benefit of telemedicine in obesity care. Obesity (Silver Spring). 2022 Mar;30(3):577-586. doi: 10.1002/oby.23382. PMID: 35195367.	no new references
Hutchesson MJ, Gough C, Müller AM, Short CE, Whatnall MC, Ahmed M, Pearson N, Yin Z, Ashton LM, Maher C, Staiano AE, Mauch CE, DeSmet A, Vandelanotte C. eHealth interventions targeting nutrition, physical activity, sedentary behavior, or obesity in adults: A scoping review of systematic reviews. Obes Rev. 2021 Oct;22(10):e13295. doi: 10.1111/obr.13295. Epub 2021 Jun 23. PMID: 34159684.	no new references
Ufholz K, Bhargava D. A Review of Telemedicine Interventions for Weight Loss. Curr Cardiovasc Risk Rep. 2021;15(9):17. doi: 10.1007/s12170-021-00680-w. Epub 2021 Jul 15. PMID: 34306296; PMCID: PMC8280385.	one new reference Houser et al. 2019
Vasselli JR, Juray S, Trasino SE. Success and failures of telehealth during COVID-19 should inform digital applications to combat obesity. Obes Sci Pract. 2021 Aug 19;8(2):254-258. doi: 10.1002/osp4.551. PMID: 34540264; PMCID: PMC8441632.	no new references

Grey literature

REGULATORY AGENCIES

The Agency for Healthcare Research and Quality (USA)

The Institute for Quality and Efficiency in Health Care (Germany)

The Institute of Medicine in the US (Institute of Medicine 2011, Agency for Healthcare Research and Quality 2014, Institute for Quality and Efficiency in Health Care 2020)

DATABASES

TROVE (Australia)

https://trove.nla.gov.au/

Keywords: obesity AND ("weight loss" OR "weight loss management" OR "weight management" Year range to: 2023 Year range from: 2018 Format: Review

no relevant studies found

Global Index Medicus

https://www.globalindexmedicus.net/

tw:(((telemedicine) OR (telehealth) OR (digital health) OR (ehealth) OR (e-health) OR (mhealth) OR (m-health) OR (web-based) OR (web based)) AND ((obesity) OR (overweight) OR (weight loss) OR (weight maintenance) OR (weight management))) AND (type_of_study:("systematic_reviews" OR "sysrev_observational_studies")) AND (year_cluster:[2018 TO 2023])

100 search results, no relevant studies

International HTA Database (INAHTA)

https://database.inahta.org/

Keywords: ((telemedicine) OR (telehealth) OR (digital health) OR (ehealth) OR (e-health) OR (mhealth) OR (m-health) OR (web-based) OR (web based)) AND ((obesity) OR (overweight) OR (weight loss) OR (weight maintenance) OR (weight management))) from 2018-2023; 46 hits, no relevant

46 search results, no relevant reviews.

Data Archiving and Network Services (DANS)

https://easy.dans.knaw.nl/ui/home

Keywords: ((telemedicine) OR (telehealth) OR (digital health) OR (ehealth) OR (e-health) OR (mhealth) OR (m-health) OR (web-based) OR (web based)) AND ((obesity) OR (overweight) OR (weight loss) OR (weight maintenance) OR (weight management))

No relevant results.

TRIAL REGISTRIES

International prospective register of systematic reviews (PROSPERO)

https://www.crd.york.ac.uk/prospero/

Keywords: obesity AND ("weight loss" OR "weight loss maintenance" OR "weight maintenance") AND (Review_Completed_not_published OR Review_Completed_published):RS AND (Systematic Review):RT

198 search results, no additional relevant ongoing or completed reviews.

Systematic Review Data Repository Plus (SRDR+)

https://srdrplus.ahrq.gov/
Keywords: ((telemedicine) OR (telehealth) OR (digital health) OR (ehealth) OR (e-health) OR (mhealth) OR (m-health) OR (web-based) OR (web based)) AND ((obesity) OR (overweight) OR (weight loss) OR (weight maintenance) OR (weight management))

No relevant results.

Table S1. List of records excluded after full text review

First author(s)	Pub year	Title	Publication	Pub date	Volume (issue)	Pages	doi	issn and isbn	Reason for exclusion	Notes
Allman-Farinelli, Margaret	2020	The efficacy of electronic health interventions targeting improved sleep for achieving prevention of weight gain in adolescents and young to middle-aged adults: A systematic review.	Obesity reviews: an official journal of the International Association for the Study of Obesity	Jun 2020	21(6)	e13006	10.1111/obr.13006	ISSN:1467-789X (Electronic)	Intervention	No weight loss intervention.
Ballin, Marcel	2020	Digital exercise interventions for improving measures of central obesity: a systematic review.	International journal of public health	Jun 2020	65(5)	593-605	10.1007/s00038-020-01385-4	ISSN:1661-8556 (Print)	Intervention	No weight loss intervention.
Bassi, G.	2021	Efficacy of ehealth interventions for adults with diabetes: A systematic review and meta-analysis	.	2021	18(17)	.	10.3390/ijerph18178982	.	Intervention	Diabetes management, not weight loss, interventions.
Benajiba, Nada	2022	Technology-based nutrition interventions using the Mediterranean diet: a systematic review	.	09 May 2022	80(6)	1419-1433	10.1093/nutrit/nuab076	0029-6643	Intervention	This is a study on interventions promoting the mediterranean diet.
Canuto, Raquel	2021	Nutritional intervention strategies for the management of overweight and obesity in primary health care: A systematic review with meta-analysis.	Obesity reviews : an official journal of the International Association for the Study of Obesity	2021	22(3)	e13143	10.1111/obr.13143	1467-789X	Intervention	No eHealth intervention.
Cavero-Redondo, Iván	2020	Effect of Behavioral Weight Management Interventions Using Lifestyle mHealth Self-Monitoring on Weight Loss: A Systematic Review and Meta-Analysis.	Nutrients	03 Jul 2020	12(7)		10.3390/nu12071977	2072-6643	Intervention	Focuses only on self-monitoring, not complete eHealth interventions.

First author(s)	Pub year	Title	Publication	Pub date	Volume (issue)	Pages	doi	issn and isbn	Reason for exclusion	Notes
Chau, Michelle M	2018	The use of social media in nutrition interventions for adolescents and young adults-A systematic review.	International journal of medical informatics	Dec 2018	120	77-91	10.1016/j.ijmedinf.2018.10.001	ISSN:1386-5056 (Print)	Intervention	Social media platforms, not eHealth interventions.
Cheatham, Scott W	2018	The efficacy of wearable activity tracking technology as part of a weight loss program: a systematic review.	The Journal of sports medicine and physical fitness	Apr 2018	58(4)	534-548	10.23736/S0022-4707.17.07437-0	ISSN:1827-1928 (Electronic)	Intervention	Wearable devices, not eHealth interventions.
Chen, D.	2020	Effect of electronic health interventions on metabolic syndrome: A systematic review and meta-analysis	.	2020	10(10)		10.1136/bmjopen-2020-036927	.	Intervention	Intervention focuses on metabolic syndrome.
Cordella, P.	2021	Coaching for the treatment of obesity and overweight: a systematic review	.	2021	149(11)	1594-1605	10.4067/S0034-98872021001101594	.	Intervention	Interventions include remote coaching (e.g. via telehphone) but are not digital interventions as specified in the inclusion criteria.
Enyioha, Chineme	2022	Effectiveness of Mobile Phone and Web-Based Interventions for Diabetes and Obesity Among African American and Hispanic Adults in the United States: Systematic Review.	JMIR public health and surveillance	04 Feb 2022	8(2)	e25890	10.2196/25890	ISSN:2369-2960 (Electronic)	Intervention	Intervention types not applicable when compared to inclusion criteria.
Fawcett, Emily	2020	Long-Term Weight Management Using Wearable Technology in Overweight and Obese Adults: Systematic Review.	JMIR mHealth and uHealth	10 Mar 2020	8(3)	e13461	10.2196/13461	ISSN:2291-5222 (Electronic)	Intervention	.
Gómez-de-Regil, Lizzette	2020	Mobile Apps for the Management of Comorbid Overweight/Obesity and Depression/Anxiety: A Systematic Review.	Journal of healthcare engineering	2020	2020	9317179	10.1155/2020/9317179	ISSN:2040-2295 (Print)	Intervention	No weight loss intervention.

First author(s)	Pub year	Title	Publication	Pub date	Volume (issue)	Pages	doi	issn and isbn	Reason for exclusion	Notes
Hangaard , S.	2021	The Effectiveness of Telemedicine Solutions for the Management of Type 2 Diabetes: A Systematic Review, Meta-Analysis, and Meta-Regression	.	2021	.	.	10.1177/193229682110646 33	.	Intervention	Interventions for type 2 diabetes, not weight loss.
Harris, Leanne	2018	The effects of multi-component weight management interventions on weight loss in adults with intellectual disabilities and obesity: A systematic review and meta-analysis of randomised controlled trials	Res Dev Disabil	Jan 2018	72	42-55	10.1016/j.ridd.2017.10.021	1873-3379	Intervention	No eHealth intervention.
Jo, Ara	2019	Is There a Benefit to Patients Using Wearable Devices Such as Fitbit or Health Apps on Mobiles? A Systematic Review.	The American journal of medicine	Dec 2019	132(12)	1394-1400.e 1	10.1016/j.amjmed.2019.06.018	ISSN:1555-7162 (Electronic)	Intervention	.
Joseph, Rodney P.	2019	A Systematic Review of Electronic and Mobile Health (e-and mHealth) Physical Activity Interventions for African American and Hispanic Women	.	Mar 2019	16(3)	230-239	10.1123/jpah.2018-0103	1543-3080	Intervention	No weight loss intervention.
Jung, J.	2022	Promoting Physical Activity and Weight Loss With mHealth Interventions Among Workers: Systematic Review and Meta-analysis of Randomized Controlled Trials	.	2022	10(1)	.	10.2196/30682	.	Intervention	Interventions target physical activity, not interventions for weight loss.
Lozano-Chacon, Blanca	2021	Use and Effectiveness of Social-Media-Delivered Weight Loss Interventions among Teenagers and Young Adults: A Systematic Review.	International journal of environmental research and public health	2021	18(16)	.	10.3390/ijerph18168493	1660-4601	Intervention	Studies social media, not the types of interventions of interest in this study.

First author(s)	Pub year	Title	Publication	Pub date	Volume (issue)	Pages	doi	issn and isbn	Reason for exclusion	Notes
Maciejewski, M.L.	2018	Systematic Review of Behavioral Weight Management Program MOVE! for Veterans	.	2018	54(5)	704-714	10.1016/j.amepre.2018.01.029	.	Intervention	.
Malkawi, A.M.	2018	Dietary, physical activity, and weight management interventions among active-duty military personnel: A systematic review	.	2018	5(1)	.	10.1186/s40779-018-0190-5	.	Intervention	No weight loss intervention.
Moghimi, Elnaz	2021	The Efficacy of eHealth Interventions for the Treatment of Adults Diagnosed With Full or Subthreshold Binge Eating Disorder: Systematic Review and Meta-analysis.	Journal of medical Internet research	20 Jul 2021	23(7)	e17874	10.2196/17874	ISSN:1439-4456 (Print)	Intervention	.
Morrill, K.E.	2021	Weight Loss Interventions for Hispanic Women in the United States: A Systematic Review	.	2021	2021	.	10.1155/2021/8714873	.	Intervention	No digital intervention.
Nitschke, Erin	2022	Impact of Nutrition and Physical Activity Interventions Provided by Nutrition and Exercise Practitioners for the Adult General Population: A Systematic Review and Meta-Analysis.	Nutrients	2022	14(9)	.	10.3390/nu14091729	2072-6643	Intervention	No weight loss intervention.
Oh, Y.J.	2021	A systematic review of artificial intelligence chatbots for promoting physical activity, healthy diet, and weight loss	.	2021	18(1)	.	10.1186/s12966-021-01224-6	.	Intervention	Studies the use of AI, no interventions of interest.
Park, SH	2019	Effect of Mobile Health on Obese Adults: A Systematic Review and Meta-Analysis	.	Jan 2019	25(1)	12-26	10.4258/hir.2019.25.1.12	2093-3681	Intervention	Interventions in focus not in line with inclusion criteria.

First author(s)	Pub year	Title	Publication	Pub date	Volume (issue)	Pages	doi	issn and isbn	Reason for exclusion	Notes
Peng, S.	2023	The Effectiveness of E-Health Interventions Promoting Physical Activity and Reducing Sedentary Behavior in College Students: A Systematic Review and Meta-Analysis of Randomized Controlled Trials	.	2023	20(1)	.	10.3390/ijerph20010318	.	Intervention	.
Petkovic, Jennifer	2021	Behavioural interventions delivered through interactive social media for health behaviour change, health outcomes, and health equity in the adult population.	The Cochrane database of systematic reviews	31 May 2021	5(5)	CD012932	10.1002/14651858.CD012932.pub2	ISSN:1469-493X (Electronic)	Intervention	.
Platini, H.	2023	Systematic Review and Meta-Analysis of Telecoaching for Self-Care Management among Persons with Type 2 Diabetes Mellitus	.	2023	20(1)	.	10.3390/ijerph20010237	.	Intervention	Telecoaching for type 2 diabetes.
Raber, Margaret	2021	A systematic review of the use of dietary self-monitoring in behavioural weight loss interventions: delivery, intensity and effectiveness	Public Health Nutr	Dec 2021	24(17)	5885-5913	10.1017/S136898002100358X	1475-2727	Intervention	Examines self-monitoring, focus not weight loss.
Sittig, S.	2020	Extensive Review of Persuasive System Design Categories and Principles: Behavioral Obesity Interventions	.	2020	44(7)	.	10.1007/s10916-020-01591-w	.	Intervention	.
Stockwell, Stephanie	2019	Digital behavior change interventions to promote physical activity and/or reduce sedentary behavior in older adults: A systematic review and meta-analysis	.	Jun 2019	120	68-87	10.1016/j.exger.2019.02.020	0531-5565	Intervention	.

First author(s)	Pub year	Title	Publication	Pub date	Volume (issue)	Pages	doi	issn and isbn	Reason for exclusion	Notes
Wong, Sai Ho	2022	Wearable technology-delivered lifestyle intervention amongst adults with overweight and obese: A systematic review and meta-regression.	International journal of nursing studies	2022	127	104163	10.1016/j.ijnurstu.2021.104163	1873-491X	Intervention	.
Xu, Y.	2021	The efficacy of mobile health in alleviating risk factors related to the occurrence and development of coronary heart disease: A systematic review and meta-analysis	.	2021	44(5)	609-619	10.1002/clc.23596	.	Intervention	.
Young, Claire	2019	Supporting Engagement, Adherence, and Behavior Change in Online Dietary Interventions.	Journal of nutrition education and behavior	Jun 2019	51(6)	719-739	10.1016/j.jneb.2019.03.006	ISSN:1878-2620 (Electronic)	Intervention	.
Zhang, Meng	2022	Efficacy of Mobile Health Applications to Improve Physical Activity and Sedentary Behavior: A Systematic Review and Meta-Analysis for Physically Inactive Individuals	.	Apr 2022	19(8)	.	10.3390/ijerph19084905	1660-4601	Intervention	Focuses on physical activity, not weight loss
Arsenijevic, Jelena	2020	Adherence to Electronic Health Tools Among Vulnerable Groups: Systematic Literature Review and Meta-Analysis.	Journal of medical Internet research	06 Feb 2020	22(2)	e11613	10.2196/11613	ISSN:1439-4456 (Print)	Outcome	.
Chatterjee, A.	2021	Digital interventions on healthy lifestyle management: Systematic review	.	2021	23(11)	.	10.2196/26931	.	Outcome	Outcome healthy lifestyle behaviours, not weight change.
Chen, Yang	2020	The Promotion of Eating Behaviour Change through Digital Interventions	.	Oct 2020	17(20)	.	10.3390/ijerph17207488	1660-4601	Outcome	Intervention for healthy eating, not weith loss.

First author(s)	Pub year	Title	Publication	Pub date	Volume (issue)	Pages	doi	issn and isbn	Reason for exclusion	Notes
Cheng, Christina	2020	Applying the Electronic Health Literacy Lens: Systematic Review of Electronic Health Interventions Targeted at Socially Disadvantaged Groups	.	13 Aug 2020	22(8)	.	10.2196/18476	1438-8871	Outcome	No weight loss intervention.
Christopoulou, SC	2018	Evidence-based health and clinical informatics: a systematic review on randomized controlled trials	.	May 2018	8(1-2)	137-150	10.1007/s12553-016-0170-2	2190-7188	Outcome	Outcome not weight change.
Cotie, L M	2018	The effectiveness of eHealth interventions on physical activity and measures of obesity among working-age women: a systematic review and meta-analysis.	Obesity reviews : an official journal of the International Association for the Study of Obesity	Oct 2018	19(10)	1340-1358	10.1111/obr.12700	ISSN:1467-789X (Electronic)	Outcome	focuses on physical activity
Dao, Kim Phuong	2021	Smartphone-Delivered Ecological Momentary Interventions Based on Ecological Momentary Assessments to Promote Health Behaviors: Systematic Review and Adapted Checklist for Reporting Ecological Momentary Assessment and Intervention Studies	.	02 Nov 2021	9(11)	.	10.2196/22890	2291-5222	Outcome	.
Duan, Y.	2021	Effects of eHealth-based multiple health behavior change interventions on physical activity, healthy diet, and weight in people with noncommunicable diseases: Systematic review and meta-analysis	.	2021	23(2)	.	10.2196/23786	.	Outcome	does not focus on weight loss

First author(s)	Pub year	Title	Publication	Pub date	Volume (issue)	Pages	doi	issn and isbn	Reason for exclusion	Notes
Gentili, A.	2022	The cost-effectiveness of digital health interventions: A systematic review of the literature	.	2022	10	.	10.3389/fpubh.2022.787135	.	Outcome	.
Hu, Ruiqi	2020	Perspectives of People Who Are Overweight and Obese on Using Wearable Technology for Weight Management: Systematic Review.	JMIR mHealth and uHealth	13 Jan 2020	8(1)	e12651	10.2196/12651	ISSN:2291-5222 (Electronic)	Outcome	.
Jeem, Yaltafit Abror	2022	The Use of Mobile Health Interventions for Outcomes among Middle-Aged and Elderly Patients with Prediabetes: A Systematic Review.	International journal of environmental research and public health	20 Oct 2022	19(20)	.	10.3390/ijerph192013638	ISSN:1661-7827 (Print)	Outcome	Outcome health behaviour and diabetes incidence, not about eHealth weight loss interventions.
Knowlden, A.P.	2022	Systematic review of electronically delivered behavioral obesity prevention interventions targeting men	.	2022	23(9)	.	10.1111/obr.13456	.	Outcome	Assessed different types of interventions available, not efficacy..
Oikonomidi, Theodora	2019	A Methodologic Systematic Review of Mobile Health Behavior Change Randomized Trials	.	Dec 2019	57(6)	836-843	10.1016/j.amepre.2019.07.008	0749-3797	Outcome	.
Patel, M.L.	2019	Motivational interviewing in eHealth and telehealth interventions for weight loss: A systematic review	.	2019	126	.	10.1016/j.ypmed.2019.05.026	.	Outcome	Studies motivational interviewing, no weight loss intervention.
Patel, Michele L	2021	Self-Monitoring via Digital Health in Weight Loss Interventions: A Systematic Review Among Adults with Overweight or Obesity.	Obesity (Silver Spring, Md.)	Mar 2021	29(3)	478-499	10.1002/oby.23088	ISSN:1930-739X (Electronic)	Outcome	.
Robert, Caroline	2021	Effectiveness of eHealth Nutritional Interventions for Middle-Aged and Older	.	17 May 2021	23(5)	.	10.2196/15649	1438-8871	Outcome	Focuses on nutrition, not weight loss

First author(s)	Pub year	Title	Publication	Pub date	Volume (issue)	Pages	doi	issn and isbn	Reason for exclusion	Notes
		Adults: Systematic Review and Meta-analysis								
Rocha, Norma Patricia Rodriguez	2019	eHealth Interventions for Fruit and Vegetable Intake: A Meta-Analysis of Effectiveness	.	Dec 2019	46(6)	947-959	10.1177/1090198119859396	1090-1981	Outcome	No weight loss intervention.
Ryan, K.	2019	A systematic review of tailored eHealth interventions for weight loss	.	2019	5	.	10.1177/2055207619826685	.	Outcome	Investigates the types and effects of tailoring, not the outcomes of weight loss interventions as specified in the inclusion criteria.
Salwen-Deremer, J.K.	2020	Incorporating Health Behavior Theory into mHealth: an Examination of Weight Loss, Dietary, and Physical Activity Interventions	.	2020	5(1)	51-60	10.1007/s41347-019-00118-6	.	Outcome	.
Scarry, Alan	2022	Usage of Mobile Applications or Mobile Health Technology to Improve Diet Quality in Adults	.	Jun 2022	14(12)	.	10.3390/nu14122437	2072-6643	Outcome	.
Sediva, Hana	2022	Behavior Change Techniques in Digital Health Interventions for Midlife Women: Systematic Review	.	Nov 2022	10(11)	.	10.2196/37234	2291-5222	Outcome	This is about BCT:s, not weight loss
Sequi-Dominguez, I.	2020	Effectiveness of mobile health interventions promoting physical activity and lifestyle interventions to reduce cardiovascular risk among individuals with metabolic syndrome: Systematic review and meta-analysis	.	2020	22(8)	.	10.2196/17790	.	Outcome	targeting cardiometabolic factors; not weight loss interventions

First author(s)	Pub year	Title	Publication	Pub date	Volume (issue)	Pages	doi	issn and isbn	Reason for exclusion	Notes
Sharkey, Thomas	2020	Effectiveness of gender-targeted versus gender-neutral interventions aimed at improving dietary intake, physical activity and/or overweight/obesity in young adults (aged 17-35 years): a systematic review and meta-analysis	Nutr J	30 Jul 2020	19(1)	78	10.1186/s12937-020-00594-0	1475-2891	Outcome	Focus not on weight loss.
Shoneye, C.L.	2022	Dietary assessment methods used in adult digital weight loss interventions: A systematic literature review	.	2022	.	.	10.1111/jhn.13101	.	Outcome	.
Sieczkowska, S.M.	2021	Health Coaching Strategies for Weight Loss: A Systematic Review and Meta-Analysis	.	2021	12(4)	1449-1460	10.1093/advances/nmaa159	.	Outcome	Outcome not weight change.
Smith, N.	2020	A systematic review of the dose-response relationship between usage and outcomes of online physical activity weight-loss interventions	.	2020	22	.	10.1016/j.invent.2020.100344	.	Outcome	.
Szinay, Dorothy	2020	Influences on the Uptake of and Engagement With Health and Well-Being Smartphone Apps: Systematic Review	.	29 May 2020	22(5)	.	10.2196/17572	1438-8871	Outcome	.
Van Rhoon, L.	2020	A systematic review of the behaviour change techniques and digital features in technology-driven type 2 diabetes prevention interventions	.	2020	6	.	10.1177/20552076209144 27	.	Outcome	Focuses on diabetes prevention, not weight loss.
Villinger, K	2019	The effectiveness of app-based mobile interventions on nutrition behaviours and nutrition-related health	.	Oct 2019	20(10)	1465-1484	10.1111/obr.12903	1467-7881	Outcome	Focuses on nutrition behaviour.

First author(s)	Pub year	Title	Publication	Pub date	Volume (issue)	Pages	doi	issn and isbn	Reason for exclusion	Notes
		outcomes: A systematic review and meta-analysis								
Willmott, Taylor	2019	Reported theory use in electronic health weight management interventions targeting young adults: a systematic review.	Health psychology review	Sep 2019	13(3)	295-317	10.1080/17437199.2019.1625280	ISSN:1743-7202 (Electronic)	Outcome	.
Wu, X.	2019	The efficacy of mobile phone apps for lifestyle modification in diabetes: Systematic review and meta-analysis	.	2019	7(1)	.	10.2196/12297	.	Outcome	.
Yang, M.	2023	Effects of Face-to-Face and eHealth Blended Interventions on Physical Activity, Diet, and Weight-Related Outcomes among Adults: A Systematic Review and Meta-Analysis	.	2023	20(2)	.	10.3390/ijerph20021560	.	Outcome	Outcome physical activity and diet.
Yang, Qinghua	2020	The Effectiveness of Interactivity in Improving Mediating Variables, Behaviors and Outcomes of Web-Based Health Interventions: A Meta-Analytic Review	.	18 Sep 2020	35(11)	1334-1348	10.1080/10410236.2019.1631992	1041-0236	Outcome	Focuses on features, not on weight loss outcomes
Ziegeldorf, Alexandra	2020	Effects of media-assisted therapeutic approaches on physical activity of obese adults: a systematic review.	BMC endocrine disorders	21 Feb 2020	20(1)	28	10.1186/s12902-020-0505-x	ISSN:1472-6823 (Electronic)	Outcome	Focuses on physical activity, not weight loss
Berry, MP	2022	Associations between behaviour change technique clusters and weight loss outcomes of automated digital interventions: a systematic review and meta-regression	.	.	.	.	10.1080/17437199.2022.2125038	1743-7199	Outcome	Investigates how to build an effective fully automated intervention using behaviour change techniques.

First author(s)	Pub year	Title	Publication	Pub date	Volume (issue)	Pages	doi	issn and isbn	Reason for exclusion	Notes
Bremer, W.	2022	Recruitment and retention in mobile application-based intervention studies: a critical synopsis of challenges and opportunities	.	2022	.	.	10.1080/17538157.2022.2082297	.	Outcomr	Studies recruitement and retention.
Asbjornsen, Rikke Aune	2019	Persuasive System Design Principles and Behavior Change Techniques to Stimulate Motivation and Adherence in Electronic Health Interventions to Support Weight Loss Maintenance: Scoping Review	.	21 Jun 2019	21(6)	.	10.2196/14265	1438-8871	Population	.
Brown, Vicki	2021	A systematic review of economic evaluations of web-based or telephone-delivered interventions for preventing overweight and obesity and/or improving obesity-related behaviors	.	Jul 2021	22(7)	.	10.1111/obr.13227	1467-7881	Population	.
Cai, Xue	2020	Mobile Application Interventions and Weight Loss in Type 2 Diabetes: A Meta-Analysis.	Obesity (Silver Spring)	Mar 2020	28(3)	502-509	10.1002/oby.22715	1930-739X 1930-7381	Population	Diabetes management, not weight loss, interventions.
Consavage Stanley, Katherine	2022	A systematic scoping review of the literacy literature to develop a digital food and nutrition literacy model for low-income adults to make healthy choices in the online food retail ecosystem to reduce obesity risk.	Obesity reviews : an official journal of the International Association for the Study of Obesity	Apr 2022	23(4)	e13414	10.1111/obr.13414	ISSN:1467-7881 (Print)	Population	No weight loss intervention.
Delva, Sabianca	2021	Efficacy of Mobile Health for Self-management of Cardiometabolic Risk Factors:	The Journal of cardiovascular nursing	2021 Jan/Feb	36(1)	34-55	10.1097/JCN.0000000000000659	ISSN:0889-4655 (Print)	Population	targeting cardiometabolic factors; not weight loss interventions

First author(s)	Pub year	Title	Publication	Pub date	Volume (issue)	Pages	doi	issn and isbn	Reason for exclusion	Notes
		A Theory-Guided Systematic Review.								
Halligan, Julia	2021	Reducing weight and BMI following gestational diabetes: a systematic review and meta-analysis of digital and telemedicine interventions	.	2021	9(1)	.	10.1136/bmjdrc-2020-002077	2052-4897	Population	Focuses on people with gestational diabetes.
Kim, Gaeun	2021	A Technology-Mediated Interventional Approach to the Prevention of Metabolic Syndrome: A Systematic Review and Meta-Analysis	.	Jan 2021	18(2)	.	10.3390/ijerph18020512	1660-4601	Population	Not targeting people with obesity or overweight. About risk factors for metabolic syndrome.
Kwon, Oh Young	2023	The Effectiveness of eHealth Interventions on Lifestyle Modification in Patients With Nonalcoholic Fatty Liver Disease: Systematic Review and Meta-analysis.	Journal of medical Internet research	2023	25	e37487	10.2196/37487	1438-8871	Population	Not all participants were obese or overweight. This is about NAFLD, not weight los.
Mertens, L.	2019	Effect of Lifestyle Coaching Including Telemonitoring and Telecoaching on Gestational Weight Gain and Postnatal Weight Loss: A Systematic Review	.	2019	25(10)	889-901	10.1089/tmj.2018.0139	.	Population	.
Messiah, S.E.	2020	Application and effectiveness of eHealth strategies for metabolic and bariatric surgery patients: A systematic review	.	2020	6	.	10.1177/2055207619898987	.	Population	.
Michaud, Tzeyu L	2021	Assessing the Impact of Telemonitoring-Facilitated Lifestyle Modifications on Diabetes Outcomes: A Systematic Review and Meta-Analysis.	Telemedicine journal and e-health : the official journal of the American Telemedicine Association	Feb 2021	27(2)	124-136	10.1089/tmj.2019.0319	ISSN:1556-3669 (Electronic)	Population	Interventions for diabetes.

First author(s)	Pub year	Title	Publication	Pub date	Volume (issue)	Pages	doi	issn and isbn	Reason for exclusion	Notes
O'Connor, M.	2018	The Efficacy and Acceptability of Third-Wave Behavioral and Cognitive eHealth Treatments: A Systematic Review and Meta-Analysis of Randomized Controlled Trials	.	2018	49(3)	459-475	10.1016/j.beth.2017.07.007	.	Population	.
Rhodes, Alexandra	2020	Exclusively Digital Health Interventions Targeting Diet, Physical Activity, and Weight Gain in Pregnant Women: Systematic Review and Meta-Analysis	.	10 Jul 2020	8(7)	.	10.2196/18255	2291-5222	Population	.
Robinson, Anna	2020	Digital and Mobile Technologies to Promote Physical Health Behavior Change and Provide Psychological Support for Patients Undergoing Elective Surgery: Meta-Ethnography and Systematic Review.	JMIR mHealth and uHealth	01 Dec 2020	8(12)	e19237	10.2196/19237	ISSN:2291-5222 (Electronic)	Population	.
Sánchez-Gutiérrez, Carmen	2022	Effectiveness of telemedicine psychoeducational interventions for adults with non-oncological chronic disease: A systematic review.	Journal of advanced nursing	May 2022	78(5)	1267-1280	10.1111/jan.15151	ISSN:1365-2648 (Electronic)	Population	.
Sung, Meekang	2022	Using an Integrated Framework to Investigate the Facilitators and Barriers of Health Information Technology Implementation in Noncommunicable Disease Management: Systematic Review.	.	20 Jul 2022	24(7)	e37338 - e37338	10.2196/37338	1438-8871	Population	.
Willmott, Taylor Jade	2019	Weight Management in Young Adults: Systematic Review of Electronic Health	Journal of medical Internet research	06 Feb 2019	21(2)	e10265	10.2196/10265	ISSN:1439-4456 (Print)	Population	About primary weight management, not about weight loss or weight loss

First author(s)	Pub year	Title	Publication	Pub date	Volume (issue)	Pages	doi	issn and isbn	Reason for exclusion	Notes
		Intervention Components and Outcomes.								maintenance. Excluded people with obesity.
Wright, Charlene	2021	Are eHealth interventions for adults who are scheduled for or have undergone bariatric surgery as effective as usual care? A systematic review.	Surgery for obesity and related diseases : official journal of the American Society for Bariatric Surgery	Dec 2021	17(12)	2065-2080	10.1016/j.soard.2021.07.020	ISSN:1878-7533 (Electronic)	Population	.
Wyse, Rebecca	2021	The Effectiveness of Interventions Delivered Using Digital Food Environments to Encourage Healthy Food Choices: A Systematic Review and Meta-Analysis	.	Jul 2021	13(7)	.	10.3390/nu13072255	2072-6643	Population	.
Xie, Li Feng	2020	Understanding Self-Guided Web-Based Educational Interventions for Patients With Chronic Health Conditions: Systematic Review of Intervention Features and Adherence	.	13 Aug 2020	22(8)	.	10.2196/18355	1438-8871	Population	.
Candelaria Martinez, Maribel	2022	Content validity of a psychological e-health program of self-control and motivation for adults with excess weight	.	Oct 2022	12(5)	.	10.1111/cob.12530	1758-8103	Study Type	.
Cheikh-Moussa, Kamila	2020	Improving Engagement Among Patients With Chronic Cardiometabolic Conditions Using mHealth: Critical Review of Reviews	.	08 Apr 2020	8(4)	.	10.2196/15446	2291-5222	Study Type	.
Chew, Han Shi Jocelyn	2021	The potential of artificial intelligence in enhancing adult weight loss: a scoping review.	Public Health Nutr	Jun 2021	24(8)	1993-2020	10.1017/S136898002100059 8	1475-2727 1368-9800	Study Type	.

First author(s)	Pub year	Title	Publication	Pub date	Volume (issue)	Pages	doi	issn and isbn	Reason for exclusion	Notes
Eicher-Miller, Heather A.	2021	Expanding the Capabilities of Nutrition Research and Health Promotion Through Mobile-Based Applications	.	May 2021	12(3)	1032-1041	10.1093/advances/nmab022	2161-8313	Study Type	.
Encantado, J.	2022	What goes on in digital behaviour change interventions for weight loss maintenance targeting physical activity: A scoping review	.	2022	8	.	10.1177/20552076221129089	.	Study Type	.
Gamble, Abigail	2020	Effects of eHealth interventions on physical activity and weight among pregnant and postpartum women and the sociodemographic characteristics of study populations: a systematic review protocol	.	Nov 2020	18(11)	2396-2403	10.11124/JBISRIR-D-19-00378	2689-8381	Study Type	.
Ghelani, Drishti P.	2020	Mobile Apps for Weight Management: A Review of the Latest Evidence to Inform Practice	.	24 Jun 2020	11	.	10.3389/fendo.2020.00412	1664-2392	Study Type	.
Gold, Natalie	2021	Effectiveness of Digital Interventions for Reducing Behavioral Risks of Cardiovascular Disease in Nonclinical Adult Populations: Systematic Review of Reviews	.	14 May 2021	23(5)	.	10.2196/19688	1438-8871	Study Type	.
Hinchliffe, N.	2022	The Potential Role of Digital Health in Obesity Care	.	2022	39(10)	4397-4412	10.1007/s12325-022-02265-4	.	Study Type	.
Hutchesson, Melinda J.	2020	Supporting women of childbearing age in the prevention and treatment of overweight and obesity: a	BMC Womens Health	23 Jan 2020	20(1)	14	10.1186/s12905-020-0882-3	1472-6874	Study Type	.

First author(s)	Pub year	Title	Publication	Pub date	Volume (issue)	Pages	doi	issn and isbn	Reason for exclusion	Notes
		scoping review of randomized control trials of behavioral interventions								
Hutchesson, Melinda J.	2021	eHealth interventions targeting nutrition, physical activity, sedentary behavior, or obesity in adults: A scoping review of systematic reviews.	Obes Rev	Oct 2021	22(10)	e13295	10.1111/obr.13295	1467-789X 1467-7881	Study Type	.
Kim, Meelim	2021	Digital Therapeutics for Obesity and Eating-Related Problems	.	Apr 2021	36(2)	220-228	10.3803/EnM.2021.107	2093-596X	Study Type	.
Marcolino, M.S.	2018	The impact of mHealth interventions: Systematic review of systematic reviews	.	2018	6(1)	.	10.2196/mhealth.8873	.	Study Type	.
Myers-Ingram, R.	2022	Outcomes following eHealth Weight Management Interventions in Adults with Overweight and Obesity from Low Socioeconomic Groups: Protocol for a Systematic Review	.	2022	11(1)	.	10.2196/34546	.	Study Type	.
Rhee, Sang Youl	2020	Present and Future of Digital Health in Diabetes and Metabolic Disease	.	Dec 2020	44(6)	819-827	10.4093/dmj.2020.0088	2233-6079	Study Type	.
Shannon, H.H.	2019	Use of Technology in the Management of Obesity: A Literature Review	.	2019	16(Fall)	1c	.	.	Study Type	.
Ufholz, K.	2021	A Review of Telemedicine Interventions for Weight Loss	.	2021	15(9)	.	10.1007/s12170-021-00680-w	.	Study Type	.
Wang, Youfa	2020	Effectiveness of Mobile Health Interventions on Diabetes and Obesity Treatment and Management: Systematic Review of Systematic Reviews	.	28 Apr 2020	8(4)	.	10.2196/15400	2291-5222	Study Type	.

First author(s)	Pub year	Title	Publication	Pub date	Volume (issue)	Pages	doi	issn and isbn	Reason for exclusion	Notes
Yin, Z.	2020	A digital health intervention for weight management for latino families living in rural communities: Perspectives and lessons learned during development	.	2020	4(8)	.	10.2196/20679	.	Study Type	.

Table S2. Studies included in each review

Author(s), publication year	Ang et al, 2021	Antoun et al, 2022	Beleigoli et al, 2019	Berry & Kassavou et al, 2021	Berry & Sala et al, 2021	Besson et al, 2020	Chew et al, 2022	Dounavi et al, 2019	Holmes et al, 2018	Houser et al, 2019	Huang et al, 2018	Islam et al, 2020	Jahangiry et al, 2021	Lahtio et al, 2022	Lau et al, 2020	LeBlanc et al, 2018 *	Lee et al, 2022	Mamalaki et al, 2022	Mata-Gonzáles et al, 2020	Novaes et al, 2022	O'Boyle et al, 2022	Podina et al, 2018	Puigdomènech et al, 2019	Rumbo-Rodriguez et al, 2020	Shi et al, 2022	Varela et al, 2021
Abdi (2015)																	x					x		x		
Adachi (2007)																									x	
Aguiar (2016)														x												
Ahn (2020)		x																								
Ahrendt (2014)										x																
Alencar (2019)																					x			x	x	
Allen(2013)		x	x	x	x			x		x		x										x		x	x	
Allman-Farinelli (2016)								x																		
Almeida (2015)																	x									
Anderson (2010)											x			x												
Apiñaniz (2019)																								x		
Appel (2011)															x	x										
Archer (2012)										x																
Aschbrenner & Naslund (2016)																				x			x			
Aschbrenner & Naslund (2016)																				x						
Ashwell (2014)																						x				
Axley (2018)					x																					

Author(s), publication year	Ang et al, 2021	Antoun et al, 2022	Beleigoli et al, 2019	Berry & Kassavou et al, 2021	Berry & Sala et al, 2021	Besson et al, 2020	Chew et al, 2022	Dounavi et al, 2019	Holmes et al, 2018	Houser et al, 2019	Huang et al, 2018	Islam et al, 2020	Jahangiry et al, 2021	Lahtio et al, 2022	Lau et al, 2020	LeBlanc et al, 2018 *	Lee et al, 2022	Mamalaki et al, 2022	Mata-Gonzáles et al, 2020	Novaes et al, 2022	O'Boyle et al, 2022	Podina et al, 2018	Puigdomènech et al, 2019	Rumbo-Rodriguez et al, 2020	Shi et al, 2022	Varela et al, 2021
Azar (2015)																						x		x	x	
Balk-Moller (2017)																	x									
Barnes (2014)																									x	
Behm-Morawitz (2016)																								x		
Bender (2017)		x																							x	
Bender (2018)	x																								x	
Bennett & Steinberg (2018)																					x					
Bennett & Warner (2012)										x																
Bhuyan (2016)								x																		
Binks (2010)																			x							
Block (2015)																							x		x	
Blomfield (2013)																										x
Blomfield (2014)																						x				
Bond (2014)																							x			
Bouhaidar (2013)					x					x														x		
Bove (2013)											x															
Brindal & Freyne (2012)																						x			x	
Brindal & Hendrie (2013)		x										x										x			x	
Brindal & Hendrie (2018)					x	x																				
Brindal & Hendrie (2019)																		x								
Brown (2014)																				x						
Bujnowska-Fedak (2011)											x															

Author(s), publication year	Ang et al, 2021	Antoun et al, 2022	Beleigoli et al, 2019	Berry & Kassavou et al, 2021	Berry & Sala et al, 2021	Besson et al, 2020	Chew et al, 2022	Dounavi et al, 2019	Holmes et al, 2018	Houser et al, 2019	Huang et al, 2018	Islam et al, 2020	Jahangiry et al, 2021	Lahtio et al, 2022	Lau et al, 2020	LeBlanc et al, 2018 *	Lee et al, 2022	Mamalaki et al, 2022	Mata-Gonzáles et al, 2020	Novaes et al, 2022	O'Boyle et al, 2022	Podina et al, 2018	Puigdomènech et al, 2019	Rumbo-Rodriguez et al, 2020	Shi et al, 2022	Varela et al, 2021
Burke & Conroy (2011)						x																			x	
Burke & Lee (2012)											x															
Burke & Styn (2012)								x		x														x		
Burke & Zheng (2017)		x																								
Cadmus-Bertram (2016)				x																						
Carnie (2013)																	x									
Carpenter (2019)																					x					
Carr (2009)													x													
Carroll (2017)								x																		
Carter (2013)		x					x	x				x											x	x	x	
Chambliss (2011)														x	x							x		x		x
Chen & Chang (2017)																				x						
Chen & Tsao (2012)														x												
Cho (2017)														x												
Choi (2019)		x																								
Christian (2011)																x										
Chung (2014)			x																		x			x		
Collins & Morgan (2012)			x	x										x	x				x		x	x				x
Collins & Morgan (2013)																									x	x
Collins & Morgan (2017)																		x							x	
Crane (2015)																			x			x			x	
Cussler (2008)									x							x		x								

Author(s), publication year	Ang et al, 2021	Antoun et al, 2022	Beleigoli et al, 2019	Berry & Kassavou et al, 2021	Berry & Sala et al, 2021	Besson et al, 2020	Chew et al, 2022	Dounavi et al, 2019	Holmes et al, 2018	Houser et al, 2019	Huang et al, 2018	Islam et al, 2020	Jahangiry et al, 2021	Lahtio et al, 2022	Lau et al, 2020	LeBlanc et al, 2018 *	Lee et al, 2022	Mamalaki et al, 2022	Mata-Gonzáles et al, 2020	Novaes et al, 2022	O'Boyle et al, 2022	Podina et al, 2018	Puigdomènech et al, 2019	Rumbo-Rodriguez et al, 2020	Shi et al, 2022	Varela et al, 2021
Damschroder (2014)																						x				
Day (2019)																	x									
Dennison (2014)																			x						x	
Devi (2014)														x												
Digenio (2009)																									x	
Donaldson (2014)									x												x					
Dong (2018)	x																									
Dorje 2019	x																									
Du (2016)								x																		
Duncan (2020)		x					x																			
Dunn & Olabode-Dada (2016)			x																							
Dunn & Turner-McGrievy							x																			
Dunn & Whetstone (2014)																								x		
Eakin (2014)														x												
Eisenhauer (2021)							x																			
Elbert (2016)								x																		
Evangelista (2018)		x																								
Faridi (2008)											x															
Farinelli (2016)												x														
Finkelstein (2015)																							x			
Fischer (2016)																x										
Fjeldsoe (2016)									x																	

Author(s), publication year	Ang et al, 2021	Antoun et al, 2022	Beleigoli et al, 2019	Berry & Kassavou et al, 2021	Berry & Sala et al, 2021	Besson et al, 2020	Chew et al, 2022	Dounavi et al, 2019	Holmes et al, 2018	Houser et al, 2019	Huang et al, 2018	Islam et al, 2020	Jahangiry et al, 2021	Lahtio et al, 2022	Lau et al, 2020	LeBlanc et al, 2018 *	Lee et al, 2022	Mamalaki et al, 2022	Mata-Gonzáles et al, 2020	Novaes et al, 2022	O'Boyle et al, 2022	Podina et al, 2018	Puigdomènech et al, 2019	Rumbo-Rodriguez et al, 2020	Shi et al, 2022	Varela et al, 2021
Forman (2014)								x																		
Fukuoka (2015)		x		x																			x		x	
Gabriele (2011)																									x	x
Garcia-Ortiz (2018)																										
Gerber (2013)										x																
Gilmore (2017)		x																							x	
Glasgow & Kurz (2010)				x																						
Glasgow & Kurz (2012)											x															
Glynn (2014)								x				x														
Godino & Golaszewski (2019)																								x		
Godino & Merchant (2016)							x									x						x		x		
Gokee (2012)																			x							
Gold (2007)						x																			x	x
Gomez-Marcos (2017)																							x			
Goulis (2004)					x																					
Green (2015)																				x						
Gregoski (2016)										x																
Gyllensten (2017)																				x						
Haapala (2009)						x								x		x						x		x		
Haddock (2014)																						x				
Hageman (2014)														x												
Hales (2016)		x			x																					

Author(s), publication year	Ang et al, 2021	Antoun et al, 2022	Beleigoli et al, 2019	Berry & Kassavou et al, 2021	Berry & Sala et al, 2021	Besson et al, 2020	Chew et al, 2022	Dounavi et al, 2019	Holmes et al, 2018	Houser et al, 2019	Huang et al, 2018	Islam et al, 2020	Jahangiry et al, 2021	Lahtio et al, 2022	Lau et al, 2020	LeBlanc et al, 2018 *	Lee et al, 2022	Mamalaki et al, 2022	Mata-Gonzáles et al, 2020	Novaes et al, 2022	O'Boyle et al, 2022	Podina et al, 2018	Puigdomènech et al, 2019	Rumbo-Rodriguez et al, 2020	Shi et al, 2022	Varela et al, 2021
Hansen (2012)														x												
Harno (2006)																										
Harrigan (2016)														x												
Hartman (2016)		x		x																						
Hartzler (2016)								x																		
Harvey-Berino & Pintauro (2004)																		x								
Harvey-Berino & Pintauro (2002)																		x								
Harvey-Berino & West (2010)						x																x			x	
Haufe (2019)		x																								
He (2017)																							x	x		
Hebden (2014)								x				x														
Hepdurgun (2020)																									x	
Hernández-Reyes (2020)					x																			x		
Holbrook (2009)											x															
Holt (2019)																				x						
Huber (2015)														x												
Hunter (2008)														x	x							x				x
Hurkmans (2018)			x			x																	x			
Hurling (2007)											x															
Hutchesson & Callister (2018)				x																						
Hutchesson & Collins (2013)																			x							
Hutchesson & Collins (2014)													x									x				

Author(s), publication year	Ang et al, 2021	Antoun et al, 2022	Beleigoli et al, 2019	Berry & Kassavou et al, 2021	Berry & Sala et al, 2021	Besson et al, 2020	Chew et al, 2022	Dounavi et al, 2019	Holmes et al, 2018	Houser et al, 2019	Huang et al, 2018	Islam et al, 2020	Jahangiry et al, 2021	Lahtio et al, 2022	Lau et al, 2020	LeBlanc et al, 2018 *	Lee et al, 2022	Mamalaki et al, 2022	Mata-Gonzáles et al, 2020	Novaes et al, 2022	O'Boyle et al, 2022	Podina et al, 2018	Puigdomènech et al, 2019	Rumbo-Rodriguez et al, 2020	Shi et al, 2022	Varela et al, 2021
Hutchesson & Morgan (2016)																			x				x			
Hutchesson & Yin (2016)																			x							
Hwang (2013)																			x							
Ing (2018)																								x		
Izquierdo (2010)											x															
Jakicic (2016)					x																			x	x	
Jane (2017)																									x	
Jarab (2012)											x															
Jebb (2011)																										
Jeffrey (2003)																						x				
Jensen (2016)																							x			
Johnston & Massey (2012)										x												x		x		
Johnston & Rost (2013)							x												x			x				
Joseph (2016)										x																
Jospe (2017)		x																								
Karhula (2015)														x												
Kaur (2020)	x																									
Keating (2019)																					x					
Kempf & Röhling (2018)																					x					
Kempf & Röhling (2019)																	x									
Kerr (2016)															x											
Kim & Faw (2017)								x		x																

Author(s), publication year	Ang et al, 2021	Antoun et al, 2022	Beleigoli et al, 2019	Berry & Kassavou et al, 2021	Berry & Sala et al, 2021	Besson et al, 2020	Chew et al, 2022	Dounavi et al, 2019	Holmes et al, 2018	Houser et al, 2019	Huang et al, 2018	Islam et al, 2020	Jahangiry et al, 2021	Lahtio et al, 2022	Lau et al, 2020	LeBlanc et al, 2018 *	Lee et al, 2022	Mamalaki et al, 2022	Mata-Gonzáles et al, 2020	Novaes et al, 2022	O'Boyle et al, 2022	Podina et al, 2018	Puigdomènech et al, 2019	Rumbo-Rodriguez et al, 2020	Shi et al, 2022	Varela et al, 2021
Kim & Kwak 2019	x																									
Kim & Oh (2015)					x																	x				
Kim & Pike (2010)										x																
Kirwan & Duncan (2012)								x																		
Kirwan & Vandelanotte (2013)								x																		
Kiselev (2012)								x																		
Kliemann (2019)																									x	
Kraschnewski (2011)			x												x							x				
Krukowski (2011)			x																			x				
Krukowsky (2013)																			x							
Kurtzman (2018)		x					x																			
Laing (2014)		x						x				x												x		
Leahey (2014)																						x				
Lee & Chae (2010)						x						x											x	x		
Lee & Cheung 2018	x																									
Lee & Kane (2014)																				x						
Lee & Kim (2016)																							x			
Lee & Kim (2019)	x																									
Lee & Shim (2019)		x																								
Lewis (2019)																								x		
Ligibel (2012)														x												
Lim & Johal 2020	x																									

Author(s), publication year	Ang et al, 2021	Antoun et al, 2022	Beleigoli et al, 2019	Berry & Kassavou et al, 2021	Berry & Sala et al, 2021	Besson et al, 2020	Chew et al, 2022	Dounavi et al, 2019	Holmes et al, 2018	Houser et al, 2019	Huang et al, 2018	Islam et al, 2020	Jahangiry et al, 2021	Lahtio et al, 2022	Lau et al, 2020	LeBlanc et al, 2018 *	Lee et al, 2022	Mamalaki et al, 2022	Mata-Gonzáles et al, 2020	Novaes et al, 2022	O'Boyle et al, 2022	Podina et al, 2018	Puigdomènech et al, 2019	Rumbo-Rodriguez et al, 2020	Shi et al, 2022	Varela et al, 2021
Lim & Kang (2011)											x															
Lim & Ong (2021)		x																								
Lin & Grambov (2018)						x																				
Lin & Mahmooth (2015)					x																					
Lin & Wang (2014)																								x		
Lisón (2020)																									x	
Little (2016)																x										
Logan (2012)								x																		
Longin (2012)																			x							
Looijmans (2019)																				x						
Lugones-Sanchez (2020)		x																								
Luley & Blaik (2011)											x															
Luley & Blaik (2014)																x										
Ma (2013)																x										
Manzoni (2016)																								x		
Mao (2017)																							x			
Margolis (2013)											x															
Martin (2015)							x								x							x	x	x		
Mason (2018)								x																		
Matthews (2006)														x												
Mattila (2013)								x																		
McCarroll (2015)																							x			

Author(s), publication year	Ang et al, 2021	Antoun et al, 2022	Beleigoli et al, 2019	Berry & Kassavou et al, 2021	Berry & Sala et al, 2021	Besson et al, 2020	Chew et al, 2022	Dounavi et al, 2019	Holmes et al, 2018	Houser et al, 2019	Huang et al, 2018	Islam et al, 2020	Jahangiry et al, 2021	Lahtio et al, 2022	Lau et al, 2020	LeBlanc et al, 2018 *	Lee et al, 2022	Mamalaki et al, 2022	Mata-Gonzáles et al, 2020	Novaes et al, 2022	O'Boyle et al, 2022	Podina et al, 2018	Puigdomènech et al, 2019	Rumbo-Rodriguez et al, 2020	Shi et al, 2022	Varela et al, 2021
McConnon (2007)			x																						x	
McDoniel (2010)													x												x	
McGrievy (2011)												x														
McKibbin (2010)																				x						
McKinstry (2013)											x															
McTigue (2009)																			x							
Mehring (2013)													x	x							x	x				
Melchart (2017)														x												
Micco (2007)																									x	
Michaelides (2016)																							x			
Monroe (2019)		x																								
Morey (2009)											x															
Morgan & Callister (2013)																						x			x	x
Morgan & Collins (2011)															x				x			x				x
Morgan & Lubans (2009)																			x			x			x	x
Morgan & Lubans (2009)				x																						
Morgan & Lubans (2010)																										x
Morgan & Lubans (2011)															x	x								x		
Morgan & Scott (2012)														x												
Morgan & Scott (2014)																			x					x		
Morrison (2014)								x																		
Mummah (2017)																							x			

Author(s), publication year	Ang et al, 2021	Antoun et al, 2022	Beleigoli et al, 2019	Berry & Kassavou et al, 2021	Berry & Sala et al, 2021	Besson et al, 2020	Chew et al, 2022	Dounavi et al, 2019	Holmes et al, 2018	Houser et al, 2019	Huang et al, 2018	Islam et al, 2020	Jahangiry et al, 2021	Lahtio et al, 2022	Lau et al, 2020	LeBlanc et al, 2018 *	Lee et al, 2022	Mamalaki et al, 2022	Mata-Gonzáles et al, 2020	Novaes et al, 2022	O'Boyle et al, 2022	Podina et al, 2018	Puigdomènech et al, 2019	Rumbo-Rodriguez et al, 2020	Shi et al, 2022	Varela et al, 2021
Muralidharan (2019)	x																									
Naimark (2015)															x								x			
Nakata (2019)																		x								
Naparstek (2017)																									x	
Napolitano (2013)						x																x		x	x	
Naslund & Aschbrenner (2016)																				x						
Naslund & Aschbrenner (2018)																				x						
Newton (2018)																								x		
Nicklas (2014)																x										
Niet (2012)									x																	
Nikolaou (2015)															x											
Nollen (2014)																							x			
O'Brien (2014)																						x				x
Oh & Cho (2015)	x																						x		x	
Oh & Kim (2003)											x															
Olson (2016)																									x	
Orsama (2013)								x																		
Ozaki (2018)																	x									
Ozaki (2019)																									x	
Pacanowski (2015)																x										
Padwal (2017)			x																							
Park (2012)								x					x													

Author(s), publication year	Ang et al, 2021	Antoun et al, 2022	Beleigoli et al, 2019	Berry & Kassavou et al, 2021	Berry & Sala et al, 2021	Besson et al, 2020	Chew et al, 2022	Dounavi et al, 2019	Holmes et al, 2018	Houser et al, 2019	Huang et al, 2018	Islam et al, 2020	Jahangiry et al, 2021	Lahtio et al, 2022	Lau et al, 2020	LeBlanc et al, 2018 *	Lee et al, 2022	Mamalaki et al, 2022	Mata-Gonzáles et al, 2020	Novaes et al, 2022	O'Boyle et al, 2022	Podina et al, 2018	Puigdomènech et al, 2019	Rumbo-Rodriguez et al, 2020	Shi et al, 2022	Varela et al, 2021
Partridge (2015)								x				x											x			
Partridge (2016)																							x**			
Patel (2019)							x					x														
Patrick & Calfas (2011)																x						x			x	
Patrick & Raab (2009)						x																x		x		
Pellegrini (2012)				x									x									x		x		
Perri (2008)											x															
Petersen (2008)																	x									
Pfaeffli (2015)								x																		
Phelan (2017)																x									x	
Polzien (2007)					x																					
Pressler (2010)																	x									
Pressman (2014)											x															
Pretlow (2015)																							x			
Pronk (2011)										x																
Purzanjani (2016)								x																		
Quintiliani (2016)																							x			
Reeves (2017)														x												
Richardson (2016)																									x	
Rimmer (2013)											x			x												
Risica (2013)											x															
Robinson (2013)								x																		

Author(s), publication year	Ang et al, 2021	Antoun et al, 2022	Beleigoli et al, 2019	Berry & Kassavou et al, 2021	Berry & Sala et al, 2021	Besson et al, 2020	Chew et al, 2022	Dounavi et al, 2019	Holmes et al, 2018	Houser et al, 2019	Huang et al, 2018	Islam et al, 2020	Jahangiry et al, 2021	Lahtio et al, 2022	Lau et al, 2020	LeBlanc et al, 2018 *	Lee et al, 2022	Mamalaki et al, 2022	Mata-Gonzáles et al, 2020	Novaes et al, 2022	O'Boyle et al, 2022	Podina et al, 2018	Puigdomènech et al, 2019	Rumbo-Rodriguez et al, 2020	Shi et al, 2022	Varela et al, 2021
Rogers (2016)		x												x										x		
Rosas (2020)							x																			
Ross (2016)		x					x																	x		
Rothert (2006)																						x			x	
Sakane (2013)														x			x									
Schroder (2010)																						x				
Shapiro (2012)						x				x						x					x	x		x		
Shaw (2013)						x																				
Sherifali (2014)																			x							
Sherwood & Jeffery (2006)																						x				
Sherwood & Jeffery (2010)										x												x				
Shigaki (2014)								x																		
Shin (2017)	x	x			x																	x				
Shuger (2011)													x	x										x		
Simpson (2017)								x																		
Smith (2014)												x														
Sniehotta (2019)																		x								
Spook (2015)																							x			
Spring & Duncan (2013)										x														x	x	
Spring & Pellegrini (2017)		x			x		x															x		x	x	
Spring & Schneider (2012)								x																		
Steinberg (2013)			x							x					x									x		

Author(s), publication year	Ang et al, 2021	Antoun et al, 2022	Beleigoli et al, 2019	Berry & Kassavou et al, 2021	Berry & Sala et al, 2021	Besson et al, 2020	Chew et al, 2022	Dounavi et al, 2019	Holmes et al, 2018	Houser et al, 2019	Huang et al, 2018	Islam et al, 2020	Jahangiry et al, 2021	Lahtio et al, 2022	Lau et al, 2020	LeBlanc et al, 2018 *	Lee et al, 2022	Mamalaki et al, 2022	Mata-Gonzáles et al, 2020	Novaes et al, 2022	O'Boyle et al, 2022	Podina et al, 2018	Puigdomènech et al, 2019	Rumbo-Rodriguez et al, 2020	Shi et al, 2022	Varela et al, 2021
Stephens (2017)		x		x						x				x								x		x		
Stuart (2012)														x												
Suen 2019	x																									
Sullivan (2013)																								x		
Sun (2016)								x																		
Svetkey & Batch (2015)		x				x		x		x						x					x	x	x	x		
Svetkey & Stevens (2008)									x							x		x								
Tanaka (2018)	x	x					x																		x	
Tang (2015)								x																		
Tate & Jackvony (2003)															x										x	
Tate & Jackvony (2006)						x									x											
Tate & Wing (2001)															x				x			x				x
Teeriniemi (2018)																									x	
Temmingh (2013)																				x						
Thomas & Bond (2019)		x																						x	x	
Thomas & Leahey (2015)										x												x				
Thomas & Raynor (2017)																x										x
Thomas & Vydelingum (2011)									x									x								
Thomas & Wing (2013)								x		x													x			
Thompson-Felty (2017)		x																								
Thorndike (2012)																		x								
Turk (2013)								x																		

Author(s), publication year	Ang et al, 2021	Antoun et al, 2022	Beleigoli et al, 2019	Berry & Kassavou et al, 2021	Berry & Sala et al, 2021	Besson et al, 2020	Chew et al, 2022	Dounavi et al, 2019	Holmes et al, 2018	Houser et al, 2019	Huang et al, 2018	Islam et al, 2020	Jahangiry et al, 2021	Lahtio et al, 2022	Lau et al, 2020	LeBlanc et al, 2018 *	Lee et al, 2022	Mamalaki et al, 2022	Mata-Gonzáles et al, 2020	Novaes et al, 2022	O'Boyle et al, 2022	Podina et al, 2018	Puigdomènech et al, 2019	Rumbo-Rodriguez et al, 2020	Shi et al, 2022	Varela et al, 2021
Turner-McGrievy & Beets (2013)								x		x															x	
Turner-McGrievy & Tate (2011)		x				x																		x		
Turner-McGrievy & Wilcox (2017)		x					x																			
Unick (2012)																								x		
van Dyck (2013)											x															
van Wier & Ariëns (2009)														x												
van Wier & Dekkers (2011)																x	x									
van Wier & Dekkers (2012)																									x	
Vaz (2018)		x																								
Wang (2012)																								x		
Watson & Bickmore (2012)										x																
Watson & Woodside (2015)				x										x											x	
Wayne (2014)								x																		
Webber (2008)																									x	
Werkman (2010)																									x	
West (2016)																					x				x	
Wharton (2014)																								x		
Whitelock (2019)		x																								
Willey (2016)																							x			
Williams (2019)																				x						
Williamson (2006)											x															
Wing (2006)									x							x		x								

Author(s), publication year	Ang et al, 2021	Antoun et al, 2022	Beleigoli et al, 2019	Berry & Kassavou et al, 2021	Berry & Sala et al, 2021	Besson et al, 2020	Chew et al, 2022	Dounavi et al, 2019	Holmes et al, 2018	Houser et al, 2019	Huang et al, 2018	Islam et al, 2020	Jahangiry et al, 2021	Lahtio et al, 2022	Lau et al, 2020	LeBlanc et al, 2018 *	Lee et al, 2022	Mamalaki et al, 2022	Mata-Gonzáles et al, 2020	Novaes et al, 2022	O'Boyle et al, 2022	Podina et al, 2018	Puigdomènech et al, 2019	Rumbo-Rodriguez et al, 2020	Shi et al, 2022	Varela et al, 2021
Womble (2004)						x																			x	x
Wong (2005)											x															
Wylie-Rosett (2001)																x									x	
Yang & Lee (2020)	x																									
Yang & Wang (2017)	x																									
Yardley (2014)			x																x		x	x				
Yoo (2009)											x															
Young & Callister (2017)																x										
Young & Cihen (2017)																				x						
Zhang (2019)	x																									
Zhou & Cheng 2016	x																									
Zhou & Zhang (2021)							x																			
Zwickert (2016)																		x								

* only studies incorporating technology

** dual publication: same study as in Partidge 2015

Table S3. Funding information of the included reviews.

Author(s), publication year	Source of funding
Ang et al, 2021	grant from the Singapore Ministry of Health's National Medical Research Council under its Health Services Research Grant program (NMRC/HSRG/0063/2016)
Antoun et al, 2022	N/A
Beleigoli et al, 2019	Study was supported with grants from the National Institute of Science and Technology for Health Technology Assessment (IATS) – CNPq/Brazil. Author AMB was was funded by Programa de Pós Doutorado (Prêmio Capes de Teses em Medicina I-2013) from Coordenação de Aperfeiçoamento de Pessoal de Nível Superior (Capes) and author ALR CNPq (research fellowship number 310679/2016–8) and FAPEMIG (PPM-00428-17).
Berry & Kassavou et al, 2021	N/A
Berry & Sala et al, 2021	N/A
Besson et al, 2020	N/A
Chew et al, 2022	grant from the National University Health System Internal Grant Funding under NUHSRO/2021/063/RO5+6/FMPCHSRG-Mar21/01
Dounavi et al, 2019	grant from MyHabeats
Holmes et al, 2018	study supported by a Postgraduate Studentship Award from the Department of the Economy (Northern Ireland)
Houser et al, 2019	supported by the National Science Foundation's Center for Healthcare Organization and Transformation (CHOT) Project 10-06181
Huang et al, 2018	no funding
Islam et al, 2020	supported by the Ministry of Education (MOE) under grant MOE 108-6604-001-400 and DP2-109-21121-01-A-01 and the Ministry of Science and Technology (MOST) under grant MOST 108-2823-8-038-002- and 109-2222-E-038-002-MY2.
Jahangiry et al, 2021	study supported by grant from research undersecretary of Tabriz University of Medical sciences (Identifier: IR.TBZMED.VCR.REC.1398.003; with the grant number of 61052).
Lahtio et al, 2022	Study supported by the Social Insurance Institution of Finland (grant number 31/26/2014)
Lau et al, 2020	N/A
LeBlanc et al, 2018	Study funded under contract HHSA290201200015I, Task Order 6, from the Agency for Healthcare Research and Quality (AHRQ), US Department of Health and Human Services, under a contract to support the USPSTF.
Lee et al, 2022	Stuy supported by the Seoul National University Hospital.
Mamalaki et al, 2022	Supported by the European Union's Horizon 2020 Research and Innovation Programme through NUTRISHIELD project (https://nutrishield-project.eu/) under Grant Agreement No. 818110.
Mata-Gonzáles et al, 2020	The National Council of Science and Technology (CONACYT) supported the academic stay in Spain of the first author.
Novaes et al, 2022	N/A

O'Boyle et al, 2022	N/A
Podina et al, 2018	Supported by a grant from the Romanian National Authority for Scientific Research and Innovation, CNCS—UEFISCDI, project number PN II-RU-TE-2014-4-2481, contract number 293/01/10/ 2015, coordinated by IRP.
Puigdomènech et al, 2019	Fully funded by the Instituto de Salud Carlos III from the Spanish Ministry of Science, Innovation and Universities, grant number PI16/01764 co-funded by FEDER.
Rumbo-Rodriguez et al, 2020	TIN2017-89069-R funded by the Ministry of Economy, Industry and Competitiveness and the European Regional Development Fund (FEDER)
Shi et al, 2022	Study supported by the Practical Research Project for Life-Style related Diseases including Cardiovascular Diseases and Diabetes Mellitus from the Japan Agency for Medical Research and Development, AMED under grant number 21ek0210124h9903.
Varela et al, 2021	Funding not applicable

Table S4. Quality of the included reviews

Author(s), publication year	1 question follows PICO framework	2 predefined potocol or guide	3 RCT/NRSI inclusion justified	4 comprehensive literature search	5 record selection in duplicate	6 data extraction in duplicate	7 provides list of excluded studies	8 describes included studies in detail	9 comprehensive RoB assessment	10 funding sources of studies reported	11 used appropriate statistical methods	12 RoB assessed in statistical analyses	13 RoB discussed	14 heterogeneity discussed	15 publication bias discussed	16 competing interests declaration	Quality of review	
LeBlanc et al, 2018	Y	PY	PY	N	Y	PY	Y	Y	Y	N	Y	Y	Y	Y	Y	Y	moderate	I3: No pre-registered protocol found. Study criteria different for efficacy and risks. I4: language restriction not justified.
Puigdomènech et al, 2019	Y	Y	Y	N	Y	Y	Y	N	Y	N	-	-	Y	Y	Y	Y	moderate	I4: English, Spanish, or French only, no justification. I3: did not explicitly explain including RCTs for one outcome and also NRSIs for another. I8: potential comparators not described.
Ang et al, 2021	Y	Y	N	N	Y	Y	N	Y	Y	N	Y	N	Y	Y	Y	Y	low	I3: RCTs, quasi-randomized trials or non-RCTs only, no justification. I4: English only, no justification.
Antoun et al, 2022	Y	N	N	PY	Y	Y	Y	Y	Y	N	Y	N	Y	Y	Y	Y	low	I3: RCTs only, no justification.
Beleigoli et al, 2019	Y	N	N	PY	Y	Y	N	Y	Y	N	Y	Y	Y	Y	Y	Y	low	I2: Did not explicitly state that there was a pre-written comprehensive protocol or guide. I3: RCT only, no justification.

Author(s), publication year	1 question follows PICO framework	2 predefined potocol or guide	3 RCT/NRSI inclusion justified	4 comprehensive literature search	5 record selection in duplicate	6 data extraction in duplicate	7 provides list of excluded studies	8 describes included studies in detail	9 comprehensive RoB assessment	10 funding sources of studies reported	11 used appropriate statistical methods	12 RoB assessed in statistical analyses	13 RoB discussed	14 heterogeneity discussed	15 publication bias discussed	16 competing interests declaration	Quality of review	
Berry & Kassavou et al, 2021	Y	Y	Y	N	Y	Y	N	Y	Y	N	Y	Y	Y	N	Y	Y	low	I4: English only, no justification.
Chew et al, 2022	Y	Y	N	N	Y	Y	N	Y	Y	Y	Y	N	Y	Y	Y	Y	low	I4: English only, not justified. Plans to use sensitivity analyses but no mention of these in results; does subgroup analysis instead.
Huang et al, 2018	Y	N	N	N	Y	N	N	Y	Y	N	Y	Y	Y	Y	Y	Y	low	I2: Claims to adhere with PRISMA guidelines, no mention of pre-defined protocol or guide. I3: RCTs only, no justification. I4: English and Chinese only, no justification.
Lahtio et al, 2022	Y	N	N	N	N	?	N	Y	Y	N	Y	Y	Y	Y	Y	Y	low	I4: Language restriction not justified. I2: Deviation from protocol not explained. Protocol states study selection to be done in duplicate, studies selected by only one reviewer.
Lau et al, 2020	Y	Y	Y	N	Y	Y	N	PY	Y	PY	Y	Y	Y	Y	Y	Y	low	I4: English only, no explanation. Study descriptions lack data on trial population sizes.
Lee et al, 2022	Y	N	N	N	Y	Y	N	Y	Y	N	-	-	Y	Y	Y	Y	low	I4: English or Korean only, not justified. Data extraction not mentioned to be in duplicate, but four reviewers assessed

Author(s), publication year	1 question follows PICO framework	2 predefined potocol or guide	3 RCT/NRSI inclusion justified	4 comprehensive literature search	5 record selection in duplicate	6 data extraction in duplicate	7 provides list of excluded studies	8 describes included studies in detail	9 comprehensive RoB assessment	10 funding sources of studies reported	11 used appropriate statistical methods	12 RoB assessed in statistical analyses	13 RoB discussed	14 heterogeneity discussed	15 publication bias discussed	16 competing interests declaration	Quality of review	
																		the tables and achieved consensus on their contents.
Mamalaki et al, 2022	Y	N	N	N	Y	Y	N	Y	Y	N	Y	N	Y	Y	Y	Y	low	I2: registered in PROSPERO but only when review was close to being finished (i.e. no preregistered protocol). I4: English only, no justification.
Podina et al, 2018	Y	N	N	PY	PY	Y	N	N	Y	N	Y	Y	Y	Y	Y	N	low	I3: RCTs only, no justification. I5: Abstracts screened by a single reviewer, full texts by two reviewers. I8: Did not describe included interventions in adequate detail.
Shi et al, 2022	Y	Y	N	N	Y	Y	N	N	Y	N	Y	N	Y	Y	Y	Y	low	I4: English or Japanese only, no justification. Did not search reference lists. I8: Did not describe interventions or controls in detail, only those in meta-analysis. I12: Did subgroup analysis but did not assess impact of RoB in analysis.
Varela et al, 2021	Y	Y	N	N	Y	Y	N	Y	Y	N	Y	Y	Y	Y	N	Y	low	I4: English and Spanish only, no justification. I2: In the protocol, the lower BMI threshold is set to 27 kg/m^2, whereas in the article it is set to 25. Deviation from protocol not explained.

Author(s), publication year	1 question follows PICO framework	2 predefined potocol or guide	3 RCT/NRSI inclusion justified	4 comprehensive literature search	5 record selection in duplicate	6 data extraction in duplicate	7 provides list of excluded studies	8 describes included studies in detail	9 comprehensive RoB assessment	10 funding sources of studies reported	11 used appropriate statistical methods	12 RoB assessed in statistical analyses	13 RoB discussed	14 heterogeneity discussed	15 publication bias discussed	16 competing interests declaration	Quality of review	
Berry & Sala et al, 2021	Y	N	PY	N	N	Y	Y	Y	Y	N	Y	Y	Y	Y	Y	Y	critically low	I3: RCT/quasi-RCT with prepost design only, no justification. I4: English only, no justification. I5: Three investigators in total screened literature but not all records were screened by at least two reviewers.
Besson et al, 2020	Y	N	N	N	Y	N	N	Y	N	N	.	.	N	Y	Y	N	critically low	I4: English only, no justification. I9 & I13: Does not assess nor discuss RoB apart from briefly mentioning publication bias in the discussion.
Dounavi et al, 2019	Y	N	N	N	Y	?	N	Y	N	N	.	.	Y	Y	.	N	critically low	I4: English only, no justification. I9: RoB not assessed in NRSIs (17 of 39 included studies). I16: Competing interest disclosed but no description of management of such interest.
Holmes et al, 2018	Y	N	N	N	Y	N	N	Y	N	N	-	-	N	N	-	Y	critically low	I3: RCTs only, no justification. I4: English only, no justification. I9: RoB not assessed.
Houser et al, 2019	Y	N	N	N	?	?	N	N	N	N	.	.	Y	Y	.	N	critically low	I4: English only, no justification. I5: Two reviewers evaluated full text studies, does not describe how the selection of records before this was conducted.

Author(s), publication year	1 question follows PICO framework	2 predefined potocol or guide	3 RCT/NRSI inclusion justified	4 comprehensive literature search	5 record selection in duplicate	6 data extraction in duplicate	7 provides list of excluded studies	8 describes included studies in detail	9 comprehensive RoB assessment	10 funding sources of studies reported	11 used appropriate statistical methods	12 RoB assessed in statistical analyses	13 RoB discussed	14 heterogeneity discussed	15 publication bias discussed	16 competing interests declaration	Quality of review	
Islam et al, 2020	Y	N	N	N	Y	?	N	Y	Y/N	N	Y/N	Y	Y	Y	Y	Y	critically low	I4: English only, no justification. I9: NRSI was not assessed for RoB. I11: NRSI included in general meta-analysis without justification and estimates were not adjusted for confounding. Separate estimates were presented only for weight, not for BMI.
Jahangiry et al, 2021	Y	Y	N	N	Y	N	N	N	N	N	Y	Y	Y	Y	Y	Y	critically low	I3: RCTs only, no explanation. I4: English only, no justification. I9: RoB assessed used the Jadad scale which is not a comprehensible RoB assessment (assesses only randomization, blinding, withdrawals, and dropouts).
Mata-Gonzáles et al, 2020	Y	N	Y	N	Y	?	N	N	N	N	.	.	Y	Y	Y	Y	critically low	I4: English or Spanish only, no justification. I8: did not describe RCT groups or sizes, did not describe interventions in adequate detail to form an overview of said interventions. I9: only assessed RoB of RCTs (11/21 of included studies).
Novaes et al, 2022	Y	N	Y	PY	Y	Y	N	N	N	N	.	.	Y	Y	.	Y	critically low	I2: Substantial deviations from protocol not explained. Protocol states maximum age of 70, review of 65. Protocol includes

Author(s), publication year	1 question follows PICO framework	2 predefined potocol or guide	3 RCT/NRSI inclusion justified	4 comprehensive literature search	5 record selection in duplicate	6 data extraction in duplicate	7 provides list of excluded studies	8 describes included studies in detail	9 comprehensive RoB assessment	10 funding sources of studies reported	11 used appropriate statistical methods	12 RoB assessed in statistical analyses	13 RoB discussed	14 heterogeneity discussed	15 publication bias discussed	16 competing interests declaration	Quality of review	
																		risk of bias and certainty of evidence assessments; these are not conducted. Data extracted deviates from protocol (e.g. does not describe phase of bipolar disorders, retention rates, intervention types, or description of interventions).
O'Boyle et al, 2022	Y	PY	N	N	N	N*	N	Y	N	N	.	.	Y	Y	.	Y	critically low	I4: English only, no justification. I9: RoB assessed using the Academy of Nutrition and Dietetics Quality Criteria Checklist, which does not assess for randomization
Rumbo-Rodriguez et al, 2020	Y	N	N	N	Y	N	N	N	N	N	-	-	N	Y	-	Y	critically low	I2: included both NRSIs and RCTs but does not justify this. I4: English and Spanish only, no justification. I6: RoB assessed in duplicate, data extraction otherwise carried out by only one reviewer. I8: Population (e.g. weight demographics) and controls (e.g. control interventions) not described in detail. I9: Assessment of RoB included in Methods, however no reports of RoB is included in the Results nor is it discussed.

RCT = Randomized Controlled Trial. NRSI = Non-Randomized Study of Intervention. RoB = Risk of Bias. Y = yes. PY = partial yes. N = no. I = item.

Quality of the included reviews was assessed using the AMSTAR-2 tool [1]. Each of the 16 items reflect potential weaknesses in quality. Items 11, 12 and 15 only apply to reviews with meta-analysis. In this study, items 2, 4, 9, 11, 13, and 15 are considered critical domains. Reviews of high quality must have no or only one non-critical weakness. Reviews of moderate quality may have more than one non-critical weakness, but multiple non-critical weaknesses may lower the appraisal to low quality. Reviews of low quality have one critical flaw with or without non-critical weaknesses. Reviews of critically low quality have more than one critical flaw with or without non-critical weaknesses.

[1] Shea BJ, Reeves BC, Wells G, Thuku M, Hamel C, Moran J, et al. AMSTAR 2: a critical appraisal tool for systematic reviews that include randomised or non-randomised studies of healthcare interventions, or both. BMJ. 2017;358:j4008.
